# Single molecule microscopy reveals diverse actions of substrate sequences that impair ClpX AAA+ ATPase function

**DOI:** 10.1016/j.jbc.2022.102457

**Published:** 2022-09-05

**Authors:** Xiao Wang, Sanford M. Simon, Philip Coffino

**Affiliations:** Laboratory of Cellular Biophysics, The Rockefeller University, New York, New York, USA

**Keywords:** ATP-dependent protease, protein degradation, protein sequence, single molecule biophysics, enzyme kinetics, BSA, bovine serum albumin, DDS, dichlorodimethylsilane, DHFR, dihydrofolate reductase, GAr, glycine-alanine repeat, LCR, low complexity region, MTX, methotrexate, polyG, polyglycine, TIRF, total internal reflection fluorescence

## Abstract

AAA+ (ATPases Associated with diverse cellular Activities) proteases unfold substrate proteins by pulling the substrate polypeptide through a narrow pore. To overcome the barrier to unfolding, substrates may require extended association with the ATPase. Failed unfolding attempts can lead to a slip of grip, which may result in substrate dissociation, but how substrate sequence affects slippage is unresolved. Here, we measured single molecule dwell time using total internal reflection fluorescence microscopy, scoring time-dependent dissociation of engaged substrates from bacterial AAA+ ATPase unfoldase/translocase ClpX. Substrates comprising a stable domain resistant to unfolding and a C-terminal unstructured tail, tagged with a degron for initiating translocase insertion, were used to determine dwell time in relation to tail length and composition. We found greater tail length promoted substrate retention during futile unfolding. Additionally, we tested two tail compositions known to frustrate unfolding. A poly-glycine tract (polyG) promoted release, but only when adjacent to the folded domain, whereas glycine-alanine repeats (GAr) did not promote release. A high complexity motif containing polar and charged residues also promoted release. We further investigated the impact of these and related motifs on substrate degradation rates and ATP consumption, using the unfoldase–protease complex ClpXP. Here, substrate domain stability modulates the effects of substrate tail sequences. polyG and GAr are both inhibitory for unfolding, but act in different ways. GAr motifs only negatively affected degradation of highly stable substrates, which is accompanied by reduced ClpXP ATPase activity. Together, our results specify substrate characteristics that affect unfolding and degradation by ClpXP.

For all organisms, AAA+ ATPase dependent proteases play an essential role in the process of protein degradation, which is critical for maintaining protein homeostasis and thus, cellular physiology. Selective protein degradation by AAA+ proteases, such as ClpXP, HslUV, and the proteasome, is necessary for various cellular activities, including clearance of misfolded proteins, removal of regulators such as cyclins and transcription factors, and presentation of antigens (1).

The bacterial protease complex ClpXP is a characteristic AAA+ protease. The complex has two particles: the unfoldase ClpX and the protease ClpP. The ring-shaped hexameric ClpX can function alone as an unfoldase; it can also dock to either or both ends of the barrel-shaped tetradecameric ClpP protease particle, to form the holoenzyme ClpXP (2). ClpX contains a narrow central pore, which is coaxially positioned with the opening of the ClpP upon docking. This architecture imposes controlled access to the proteolytic sites inside the ClpP complex. Substrates are targeted to the central pore of ClpX by their degradation signals, and substrate entry is initiated from N- or C-terminal unstructured regions (2). The 11 amino acid ssrA tag (AANDENYALAA) is one of the commonly studied degradation signals for ClpX. The tag is at the terminus of the C-terminal unstructured region of the substrate and is recognized and engaged by structural elements of the ClpX central pore (3). In this study, the entire C-terminal unstructured region including the ssrA tag is referred to as the substrate tail.

As the coaxial pores of unfoldase and protease modules are too narrow to allow passage of folded protein domains into the proteolytic chamber of ClpP, such substrates require unfolding and translocation by ClpX for degradation. This process is carried out by sets of mobile loops inside and above the ClpX central pore: RKH, pore-1, and pore-2 loops (3, 4, 5, 6). Among these, the pore-1 loops, containing the conserved GYVG motif, have been shown to be the most critical. The pore-1 loops are arranged as a spiral staircase around the substrate polypeptide, with the GYVG motif making direct physical contact with the substrate (5, 6, 7). To propel the engaged polypeptide, ClpX hydrolyzes ATP to make directional power strokes (8, 9). The force from the power stroke needs to be transduced to the substrate. Consequently, the efficiency of the unfolding process is strongly influenced by the strength of interaction between the pore-1 loops and the engaged polypeptide. This interaction we refer to as the "grip" by ClpX.

At the single molecule level, it has been shown that a folded substrate domain is sterically excluded by the central pore, resisting translocation by ClpX until it is unfolded (8, 9, 10). The period of stalled substrate processing occupied by unfolding trials is referred to as the preunfolding dwell (Fig. 1*A*), which is the rate limiting step in the degradation process and can last 5 to 50 s, depending on the substrates and temperature (11, 12). At this stage, multiple cycles of ATP hydrolysis by ClpX are required for a successful unfolding event (11, 12, 13), suggesting that ClpX mounts multiple attempts at unfolding during the preunfolding dwell. However, substrates also have a high probability of slippage from ClpX during preunfolding dwell (9, 14), which may in turn increase the rate of substrate dissociation. Therefore, whether a substrate is fully processed or prematurely released depends on the balance between unfolding and slippage.

**Figure 1:**
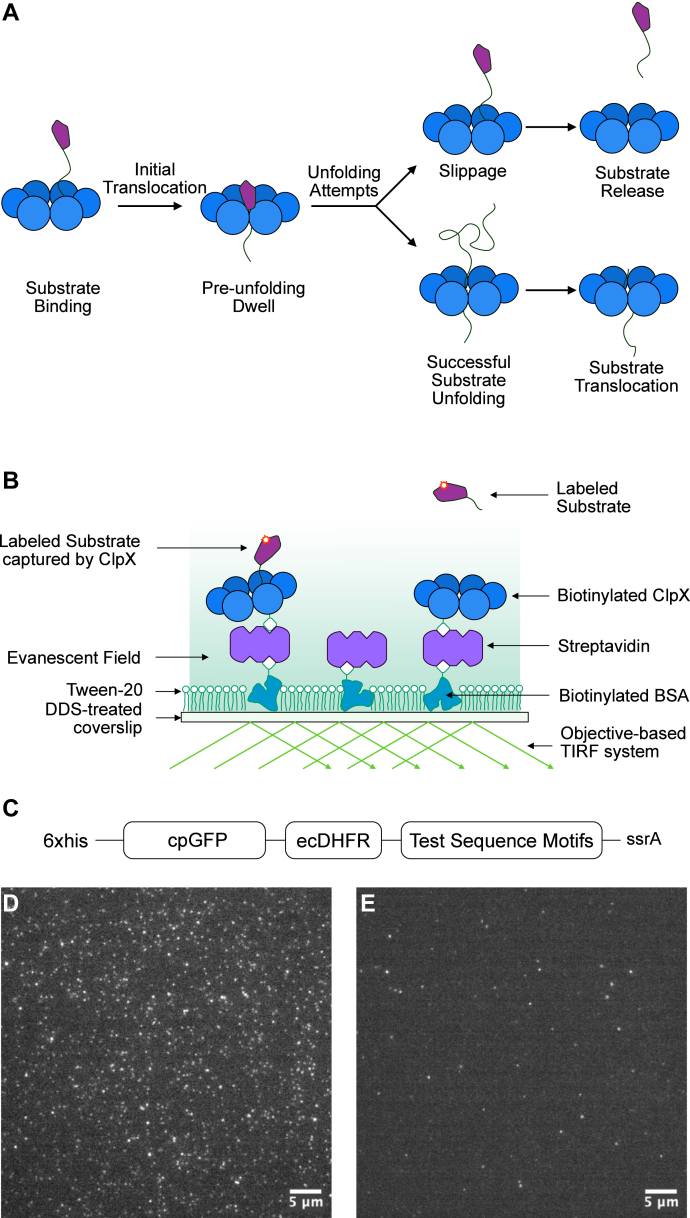
**Design of the TIRF binding assay**. *A*, schematic representation of key stages of substrate unfolding by ClpX. Upon engagement with ClpX, there are two outcomes that can lead to disengagement of the substrate: a failure of unfolding and slippage that causes retrograde release of the substrate or successful unfolding of the substrate and its processive full-length passage through the ATPase ring. *B*, schematic of the TIRF assay immobilization strategy. Coverslips were made hydrophobic with DDS. Biotinylated BSA was immobilized by nonspecific binding. The coverslip was then passivated with Tween-20. ClpX hexamers, each with a single biotinylation site, were anchored to biotinylated BSA *via* streptavidin. *C*, schematic of DHFR substrates used for TIRF microscopy (N terminus to left). Insertion and translocation are initiated from the C terminus at the ssrA degron. An *E. coli* DHFR domain was positioned adjacent to test sequences. The DHFR domain can be stabilized with MTX to resist ClpX unfolding. A cpGFP domain was placed N-terminal to the DHFR domain. Test sequence motifs of diverse sequence composition and/or length were placed between the DHFR domain and the ssrA tag. *D* and *E*, representative TIRF images for substrates containing ssrA tag and ssrADD mutant tag, respectively. The scale bar represents 5 microns. DDS, dichlorodimethylsilane; DHFR, dihydrofolate reductase; MTX, methotrexate; TIRF, total internal reflection fluorescence.

While substrate slippage is one of the crucial aspects of degradation processivity, factors that affect slippage are not well understood. Substrates with long terminal unstructured tails have been shown to promote ClpXP degradation efficiency (15), but this factor has not been systematically examined. The strength of grip over the substrate by ClpX has also been implicated in substrate slippage. Weakening the grip, either by mutating the conserved tyrosine residues in pore-1 loops or by presenting polyglycine (polyG) tracts to the pore-1 loops during unfolding, results in reduced substrate degradation rate (16, 17, 18, 19). In the former case, pore-1 loop mutations in ClpX have been reported to increase substrate slippage in optical traps experiments (14). In the latter case, it has been proposed that low complexity sequence motifs can promote substrate slippage or prevent force transduction from pore-1 loops of AAA+ proteases (20, 21, 22, 23). Although several substrate sequence motifs have been reported to reduce grip, their effects on slippage are not fully understood (19, 21, 22, 24, 25, 26).

Here, we characterize the effects of substrate tail length and sequence on the retention of substrate by ClpX at preunfolding dwell using total internal reflection fluorescence (TIRF) microscopy. In this assay, unfolding is stalled by using a structurally stable substrate—degradation of which by ClpXP is experimentally undetectable—such that the substrate can only bind and dissociate, but not unfold. This strategy simplifies the kinetic scheme of the process, thereby facilitating the analysis of the TIRF microscopy data. We find that substrates with a longer tail are retained longer by ClpX. We then evaluated the effects of sequence motifs in the substrate tail implicated to interfere with grip by pore-1 loops. We found that a polyG motif shortens the dwell time of the substrate, most effectively when positioned adjacent to the folded domain. In contrast, a glycine-alanine repeat (GAr) increases the substrate dwell time. Unexpectedly, a high complexity tail is also poorly retained by ClpX. Finally, to investigate the correlation between substrate retention and substrate degradation rate, we test the impact of the tail sequence motifs on degradation by ClpXP of substrates with different domain stability and on the ATPase activity of ClpX and ClpXP. Our results show low complexity sequences like polyG and GAr motifs exert their effects on substrate degradation by different mechanisms, and the magnitude of their effects depends on substrate domain stability. Our study illuminates the mechanisms of substrate slippage and escape from AAA+ ATPase proteases.

## Results

### Measuring persistence of substrate binding using TIRF microscopy

We used TIRF microscopy to measure the dwell time of substrates captured by ClpX. We adopted a strategy using a covalently linked hexamer of ClpX^ΔN^ containing a single biotinylation site (ClpX_6B_) (27), which was tethered by streptavidin to biotinylate bovine serum albumin (BSA) decorated on dichlorodimethylsilane (DDS)-Tween-20 passivated coverslips (28). The single chain ClpX hexamer has been characterized biochemically and structurally and shows similar enzymatic activities as homohexamer ClpX (5, 27). The unique biotinylation site on the single chain ClpX prevents excessive biotinylation of the ClpX hexamer, reducing the chance of undesirable crosslinking of ClpX hexamers by the multivalent streptavidin. In this setup, fluorophore-labeled protein substrate was added, and molecules bound to tethered ClpX were immobilized *via* their interaction with ClpX. Under TIRF illumination, labeled substrates captured by ClpX on the coverslip are selectively imaged as individual puncta, while free substrates that are predominantly outside of the evanescent excitation field do not produce strong and persistent fluorescent signals and are hence excluded from analysis (Fig. 1*B*).

Several tests were performed to confirm that TIRF signal represented specific binding of substrates to a biotinylated ClpX that was immobilized on the coverslip *via* streptavidin. First, the passivation of the coverslip was tested by assaying fluorophore-labeled streptavidin nonspecifically bound to the coverslip, in the absence of immobilized biotinylated BSA (Fig. S1). Second, to test whether the ssrA-degron–tagged substrate was associating with the coverslip through ClpX, the association of ClpX with the coverslip was blocked with an excess of free biotin (Fig. S2). Third, to determine whether fluorescent puncta observed by TIRF microscopy represent authentic interactions between ClpX and substrates, a negative control substrate was utilized, in which the C-terminal Ala-Ala residues of the ssrA degron were mutated to Asp-Asp. This modification, referred to as the ssrADD tag (AANDENYALDD), has been shown to abolish substrate targeting to ClpX (29, 30). For a mixture of ssrA or ssrADD-tagged substrates labeled with different fluorophores, the ssrA-tagged substrate was preferentially captured by immobilized ClpX. (Fig. S3). Substrate concentration was adjusted to achieve <1 punctum per μm^2^ density to avoid crowding of signals in the microscopy field, facilitating data processing by a custom processing script. Furthermore, concurrent binding events within 1 μm of each other were also excluded from dwell time distribution analysis. A representative image comparing the capture of ssrA and ssrADD-tagged substrates by ClpX is shown in Figure 1, *D* and *E*.

Substrate binding with ClpX could be terminated either by dissociation after a slippage or by unfolding and translocation of the full length of substrate through the pore. We precluded substrate unfolding by utilizing *Escherichia coli* dihydrofolate reductase (DHFR), which when stabilized with the pseudosubstrate methotrexate (MTX), is undegradable by ClpXP (31). Thus, the ssrA-tagged DHFR(MTX) cannot be translocated across the ClpX central pore; the only available path to termination of substrate binding is by disengagement. In this kinetic scheme, the rate of substrate dissociation can be described by a single rate constant. This allows the lifetime of the substrate–ClpX complex to be modeled as a single exponential decay process.

Substrate binding and dissociation is observed by the appearance and disappearance of puncta in TIRF microscopy. Fig. S4 shows the data processing steps for extracting puncta dwell time, and representative puncta trajectories are shown in Fig. S5. The distribution of dwell times for the binding events is transformed into an empirical cumulative distribution function, which can be fitted using the cumulative density function of exponential distribution to derive the average dwell time (*τ*). For each of the dwell time distributions in this study, the curve was fitted with r^2^ > 0.97.

ClpX has been observed to initiate substrate unfolding from either the C or N terminus (32). To ensure that we were observing the effects of ClpX engaging the designated C terminal tail region, rather than other substrate regions spatially close to the central pore, we attached a circular-permutated GFP (cpGFP) domain to the N terminus of the DHFR domain (Fig. S6). Any N-terminal engagement would result in the degradation of the cpGFP domain first. When MTX-bound DHFR containing N-terminal cpGFP domain was incubated with ClpXP, the full-length substrate was not degraded (Fig. S7). This confirms that the MTX-bound DHFR domain cannot be unfolded and processed by ClpXP and that unfolding and degradation cannot initiate at the N terminus. Separately, we confirmed that MTX, on its own, does not interfere with the activity of ClpXP (Fig. S7). The MTX-bound cpGFP-DHFR substrates are simply referred to as DHFR substrates in the study.

It was important to validate that the exponential decay lifetime of fluorescent puncta represented dwell times of substrate at ClpX and not photobleaching. This was tested by altering the duration of laser exposure at each time point. There was no change in the dwell distributions despite a threefold increase in total laser exposure time over the standard condition for data collection, confirming that punctum decay does not represent photobleaching (Fig. S8).

With this experimental design, the effects of substrate tail length and polypeptide amino acid sequence on the dwell time of the substrate were assessed.

### Substrate tail length affects dissociation rate

In optical trap experiments, substrate was observed to slip back when ClpX momentarily lost grip; before ClpX re-engages its grip, the distances of slippage peaked at a distance corresponding to an extended polypeptide between 30 and 40 aa in size (9). After a slippage event, a substrate with an inserted tail longer than the average slippage distance would likely allow ClpX to re-establish grip before complete dissociation of the substrate. In this view, a slippage event longer than the length of engaged polypeptide will cause dissociation.

**Figure 2:**
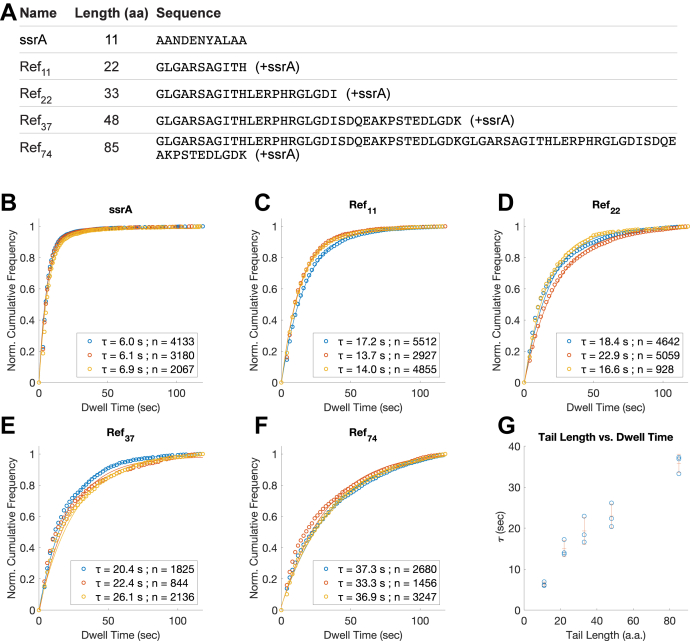
**Dwell time distributions of DHFR substrates with C-terminal tails of variable length.***A*, amino acid sequence of the tail region of substrates tested. Ref_11_ and Ref_22_ were created from the native C-terminal unstructured regions of TitinI27 domain. Ref_37_ was derived from Ref_22_ by an additional C-terminal extension, using sequence motifs from the N-terminal unstructured regions of SUMO1 protein. Ref_74_ was derived from Ref_37_ by repeating the motif. *B–F*, fitted dwell time distributions and the averages of dwell time (*τ*) for DHFR substrates with different tail lengths, as well as the number of events (n) used for deriving the dwell time. The tail length constitutes the length of the test sequence motifs, plus 11 amino acids of the ssrA tag. Three biological repeats were conducted for each substrate construct, shown here overlayed. Dwell time distribution was fitted using a single exponential decay model. All fitted curves have r^2^ > 0.97. *B*, distributions for 11 aa tail. *C*, distributions for 22 aa tail. *D*, distributions for 33 aa tail. *E*, distributions for 48 aa tail. *F*, distributions for 85 aa tail. *G*, the fitted values of *τ*, as well as the mean (±S.D.) plotted against tail length of the substrate. DHFR, dihydrofolate reductase.

To test whether the substrate tail length affects the dwell time of DHFR substrates, we designed a series of test sequences of different lengths attached to the C terminus of the DHFR substrate (Fig. 2*A*). The TIRF measurements largely confirmed the expectation that longer tail length increases dwell time (Fig. 2, *B*–*G*). The result suggests that with a fixed frequency of slippage, dissociation rate will still vary due to the recoverable slippage distance dictated by the tail length. In this model, a stronger correlation between substrate dissociation rate and slippage rate occurs when the substrate tail is short. In consideration of this effect, we varied tail amino acid composition, testing sequences previously shown to augment or impair degradation or slippage while holding fixed the length of these sequence motifs.

### polyG sequence makes the substrate more slippery

**Figure 3:**
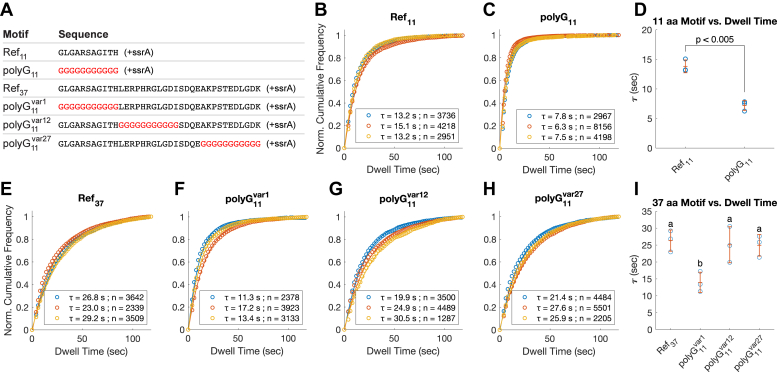
**Dwell time distributions of DHFR substrates containing polyG**_**11**_**motifs in the tail region.***A*, the amino acid sequence of the tail region for substrates containing polyG_11_ motifs, denoted in *red*. Ref_11_ and Ref_37_ were described in Figure 2*A*. polyG_11_^var1^, polyG_11_^var12^, and polyG_11_^var27^ were created by replacing the existing sequence of Ref_37_ with polyG_11_ motif at various positions. *B* and *C* and *E–H*, fitted dwell time distributions, the averages of dwell time (*τ*), and the number of events (n) used for deriving the dwell time. Three biological replicates were conducted for each substrate protein, shown here overlayed. All fitted curves have r^2^ > 0.98. *B*, distributions for Ref_11_. *C*, distributions for polyG_11_. *D*, comparison of the fitted values of *τ* for 11 aa sequence motifs and the means (±S.D.); *p* < 0.05, using two-sample *t* test. *E*, distributions for Ref_37_. *F*, distributions for polyG_11_^var1^. *G*, distributions for polyG_11_^var12^. *H*, distributions for polyG_11_^var27^. *I*, comparison of the fitted values of *τ* and the means (±SD) for 37 aa sequence motifs; *p* < 0.05 for one-way ANOVA test. Dwell times were then compared in pairwise fashion using Tukey’s honestly significant difference procedure. Dwell times cluster into two levels, as denoted by the letters (a and b); among these, only polyG_11_^var1^ had a significantly different mean (*p* < 0.05). DHFR, dihydrofolate reductase; polyG, polyglycine.

A 12 amino acid repeat sequence of polyG has been found to interfere with substrate degradation by ClpXP (19). We tested the effect of an 11 amino acid polyG cassette (polyG_11_) on dwell time. Considering the effect of tail length on substrate dissociation, we also created three test sequences in which the polyG_11_ was positioned within a longer tail. For the longer tail, we used the Ref_37_ as described in Figure 2*A*, which has higher sequence complexity than a polyG, as a template. The polyG_11_ tract was used to replace Ref_37_ starting at positions 1 (polyG_11_^var1^), 12 (polyG_11_^var12^), or 27 (polyG_11_^var27^) within the 37 amino acid test sequence (Fig. 3*A*).

Compared to Ref_11_, polyG_11_ led to a reduction of about 50% in substrate dwell time with ClpX (Fig. 3, *B*–*D*). This supports a model whereby slippage events are more common for the polyG_11_ substrate. Compared to Ref_37_, the polyG tract in the polyG_11_^var1^ led to similarly reduced substrate dwell time with ClpX (Fig. 3, *E*, *F*, and *I*). For polyG_11_^var12^ and polyG_11_^var27^, which are of the same length as polyG_11_^var1^, the average dwell times were increased to the same level as Ref_37_, indicating a reduction in slippage frequency compared to polyG_11_^var1^ (Fig. 3, *E*, *F*, *G* and *H*). These results, summarized in Figure 3, *D* and *I*, show that a polyG_11_ motif placed adjacent to the DHFR folded domain (as in polyG_11_^var1^) leads to higher chances of substrate slippage. However, polyG_11_ tracts positioned at more distal portions of the tail do not affect the slippage frequency. Thus, a key parameter of slippage is the proximity of the slippery motif to the region that is difficult to unfold.

### GArs lead to a long dwell time

**Figure 4:**
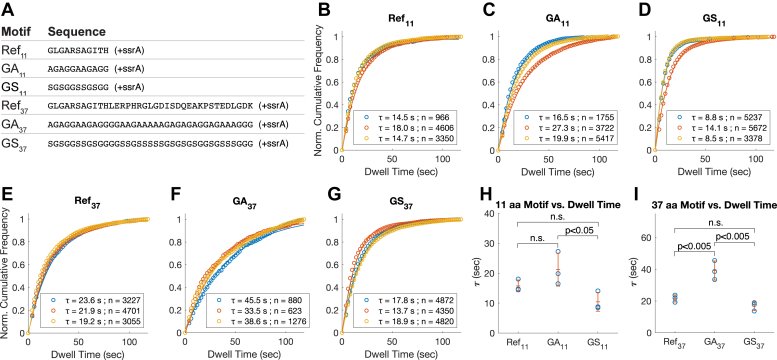
**Dwell time distributions of DHFR substrates containing GAr or GSr motifs in the tail region**. *A*, the amino acid sequence of the tail region of substrates containing GAr or GSr motifs. The alanine or serine residues were randomly positioned within the sequence motif; about 50% of residues were glycine. *B–G*, fitted dwell time distributions, the averages of dwell time (*τ*) and the number of events (n) used for deriving the dwell time. Three biological repeats were conducted for each substrate protein, shown here overlayed. All fitted curves have r^2^ > 0.98. *B*, distributions for Ref_11_. C, distributions for GA_11_. *D*, distributions for GS_11_. *E*, distributions for Ref_37_. *F*, distributions for GA_37_. *G*, distributions for GS37. *H,* the fitted values of *τ* and the means (±SD) for 11 aa sequence motifs; *p* < 0.05 for one-way ANOVA test. In pairwise comparisons, GA_11_ and GS_11_ were found to be significantly different from each other (*p* < 0.05) using Tukey’s honestly significant difference procedure. *I*, comparison of the fitted values of *τ* and the means (±SD) for 37 aa sequence motifs; *p* <0.05 for one-way ANOVA test. In pairwise comparisons, GA_37_ had a mean significantly greater than both Ref_37_ and GS_37_ (*p* < 0.05) using Tukey’s honestly significant difference procedure. DHFR, dihydrofolate reductase; GAr, glycine-alanine repeat; GSr, glycine-serine repeat.

ClpX has been reported to be faster at unfolding GFP substrates with tails that contain amino acids with bulky or hydrophobic sidechains, compared to tails containing polar and charged residues (19). However, substrate tails containing hydrophobic GAr motifs have also been shown to obstruct degradation by AAA+ proteases in certain substrates (21, 22, 24, 25). To better understand the cause of these seemingly conflicting outcomes, we investigated whether GAr motifs were slippery using TIRF dwell time analysis. We first created an 11 aa test sequence containing five alanine residues and six glycine residues (GA_11_). To assess the role of hydrophobicity in the slipperiness of GA_11_, a glycine-serine test sequence (referred to as GS_11_) was made by replacing the five alanine residues of GA_11_ with serine residues. Some naturally occurring GAr sequences such as those of the Epstein-Barr virus Nuclear Antigen 1 (EBNA1) protein are of great length, up to 300 aa (33). Considering the effects of substrate tail length, we extended the GAr from 11 aa to 37 aa to make the GA_37_ test sequence. Likewise, a corresponding GS_37_ was made by replacing alanine residues of GA_37_ with serine residues (Fig. 4*A*).

The DHFR substrates retained *via* GA_11_ had a *τ* slightly greater than that of Ref_11_ (Fig. 4, *B*, *C*, and *H*), although the difference was not statistically significant. Consequently, GA_11_ by this measure cannot be considered a slippery sequence relative to Ref_11_. While polyG and GAr sequences are both reported to interfere with ATPase unfolding, the former is more slippery than the latter, as signified by its shorter dwell time. In contrast to GA_11_, *τ* for the substrate with GS_11_ tail was slightly lower than that with Ref_11_ (Fig. 4, *D* and *H*), and GS_11_ substrates have a significantly shorter dwell time than GA_11_ substrates (Fig. 4*H*). For both the GA_11_ and GS_11_ sequences, extending the length of the tail increased the values of *τ*, consistent with the slippage distance model (Fig. 4, *F* and *G*). However, while the dwell time of GS_37_ was still slightly shorter than that of Ref_37_, GA_37_ produced a significantly longer dwell time than did Ref_37_ (Fig. 4*I*). The latter result indicates an additional length-dependent effect of GAr unexplained by the slippage distance, perhaps associated with its hydrophobicity.

### A slippery high complexity sequence

Regions of low sequence complexity (sometimes termed low complexity regions, or LCRs), have been proposed to predict slipperiness for AAA+ proteases (20, 23). To compare sequence complexity, we utilized the composition complexity score defined in the SEG algorithm (34, 35) and applied them to all the test sequence motifs used in this study (complexity scores listed in Table S1). In this system, sequences like polyG that merely repeat a single amino acid have a score of zero, while a score exceeding 2.5 is algorithmically determined to be of “high complexity” (34).

GS_11_ (SGSGGSSGSGG) and GA_11_ (AGAGGAAGAGG) are both low complexity sequences (complexity = 0.99 for both), but the latter leads to better substrate retention on ClpX, implying a preference for hydrophobicity by ClpX. Despite its high complexity, Ref_11_ (GLGARSAGITH, complexity = 2.85) has a similar retention time as the lower complexity sequence GA_11_. Because 7 out of 11 residues in Ref_11_ are hydrophobic, it is unclear whether its long dwell time is attributed to its greater hydrophobicity or its complexity. We therefore also tested an alternative 11 aa high complexity sequence based on the N-terminal unstructured region of human SUMO1 protein (AKPSTEDLGDK, complexity = 3.10), of which the compositional complexity is slightly higher than Ref_11_ but contains more polar and charged residues.

**Figure 5:**
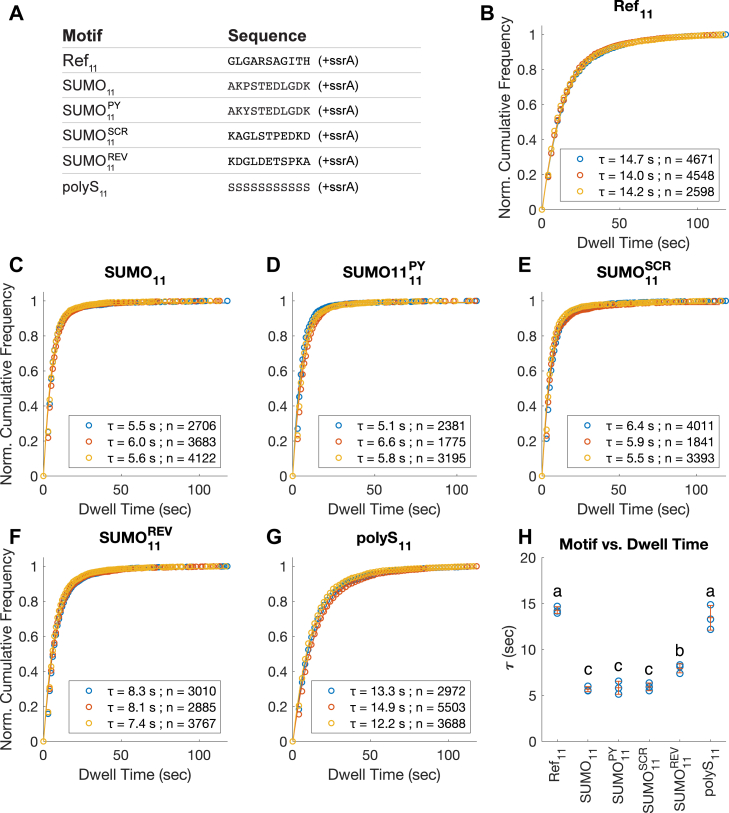
**Dwell time distributions of DHFR substrates containing SUMO11 and related motifs in the tail region.***A*, the amino acid sequence of the tail region of the tested substrates. SUMO_11_ originated from the N-terminal unstructured region of human SUMO1 protein. SUMO_11_^PY^ contains the P to Y mutation at the third position of the SUMO_11_ motif. SUMO_11_^SCR^ and SUMO_11_^REV^ were created by reshuffling the SUMO_11_ sequence in alternative ways. *B–G*, fitted dwell time distributions, the averages of dwell time (*τ*) and the number of events (n) used for deriving the dwell time. Three biological repeats were conducted for each substrate protein, shown here overlayed. All fitted curves have r^2^ > 0.99. *B*, distributions for Ref_11_. *C*, distributions for SUMO_11_. *D*, distributions for SUMO_11_^PY^. *E*, distributions for SUMO_11_^SCR^. *F*, distributions for SUMO_11_^REV^. *G*, distributions for polyS_11_. *H*, the fitted values of *τ* and the means (±SD) plotted against the identities of tail sequences; *p* < 0.05 for one-way ANOVA test. Pairwise comparisons were done using Tukey’s honestly significant difference procedure. Dwell times cluster into three levels, as denoted by the letters (a–c); among these, Ref_11_ and polyS_11_ had greater means than SUMO_11_ and the related SUMO_11_ motifs (*p* < 0.005); between the four SUMO_11_ motifs, only SUMO_11_^REV^ had significantly different mean (*p*< 0.05). DHFR, dihydrofolate reductase..

Surprisingly, the dwell time of DHFR with SUMO_11_ tail was significantly reduced compared to the dwell time of Ref_11_.(Fig. 5, *B* and *C*). What could explain the slippery property of SUMO_11_? It contains a proline residue, which might play an outsized role in disrupting pore-1 loop interactions by imposing a *cis*-peptide bond on the polypeptide backbone within the narrow central pore of ClpX. We therefore also tested the SUMO_11_^PY^ sequence, in which proline in SUMO_11_ was mutated to tyrosine. This modification did not noticeably alter the dwell time, indicating that the presence of the proline was unlikely to be a factor (Fig. 5*D*).

While the pore-1 loop spiral staircase spans the 12 amino acid region immediately adjacent to the folded domain, this region has been reported to contribute to unfolding in an asymmetrical manner, with the amino acid residues at third to fifth positions most critical for producing grip (5, 19). To test whether the slipperiness of the SUMO_11_ tail is due to the overall composition of the SUMO_11_ or the particular positioning of individual residues, we reshuffled the sequence in two ways (Fig. 5*A*): first by freely rearranging the same 11 amino acids (referred to as "SUMO_11_^SCR^") and then by reversing the sequence ("SUMO_11_^REV^"). The reversed SUMO_11_ had oppositely oriented charge polarity compared to the original, while in the scrambled SUMO_11_, the nonpolar residues, other than the proline residue, were placed at position 2 to 4. Of the two, only SUMO_11_^REV^ showed a small but significant increase in dwell time, which was still shorter than that of Ref_11_ (Fig. 5, *E*, *F*, and *H*). All three SUMO_11_-related sequence motifs have the same complexity score as SUMO_11_.

The results indicate that the composition of SUMO_11_ is the primary cause of its slipperiness. In addition to the proline residue, the SUMO_11_ sequence contained several charged and polar residues, but these properties have not been previously reported to negatively affect substrate degradations in a high complexity setting. To test whether multiple polar residues could increase slipperiness, we created an 11 amino acid polyS_11_ test sequence containing only serine residues. The dwell time of DHFR with polyS_11_ tail was similar to that of Ref_11_ (Fig. 5*G*). This indicates that polar residues, when present in sufficient quantity, can confer substrate retention as effectively as the reference sequence. Consequently, the poor retention of the SUMO_11_ cannot be explained by the presence of polar residues alone.

### Assessing the impact of test sequence on substrate degradation

**Figure 6:**
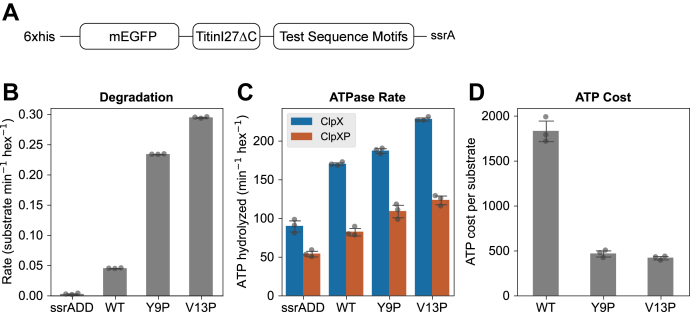
**Characterization of the TitinI27ΔC substrate, with a ssrA-only C-terminal tail**. *A*, schematic drawing of the TitinI27ΔC substrates used for characterizing test sequence motifs in bulk solution assays (N terminus to left). The overall design follows that of DHFR substrates used for TIRF microscopy, depicted in Figure 1*C*. The substrate is targeted to ClpX by the C-terminal ssrA degron. The mEGFP domain is used for monitoring substrate degradation by fluorescence. The TitinI27ΔC domain can be tuned by Y9P or V13P mutation to create lower stability variants. The test sequence motif is inserted between the TitinI27ΔC domain and the ssrA tag. *B*, degradation rates of the three variants of TitinI27ΔC substrates with ssrA-only tail. The substrate containing a ssrADD mutant degron, which ClpX does not recognize, provides a negative control. Three biological replicates were measured for each protein construct. Error bars represent SD. *C*, the ATPase rate for ClpX and ClpXP processing three variants of the TitinI27ΔC substrates with ssrA-only tail. The basal levels of ATPase activity for ClpX and ClpXP were established by incubating ClpX or ClpXP with a TitinI27ΔC substrate containing an ssrADD tag. *D*, the ATP cost of substrate degradation, calculated as the ratio of ATP hydrolysis rate divided by substrate degradation rate, for three variants of TitinI27ΔC substrate. DHFR, dihydrofolate reductase; TIRF, total internal reflection fluorescence.

The TIRF results show that substrate tail sequence adjacent to the folded domain plays an important role in the retention of substrate. We next characterized the same tail sequences for their effects on substrate degradation. However, substrates with different structured domain stability have different force thresholds for unfolding, and it is possible that a slippery sequence is permissive for unfolding certain substrates but not others. To investigate the potential interaction between sequence slipperiness and substrate stability, we utilized the TitinI27 domain to challenge the test sequences against varying loads. The load variation is achieved by tuning the stability of the TitinI27 domain using well-characterized point mutations; these lower the force threshold for TitinI27 unfolding (12, 36). We truncated the native C-terminal unstructured region of TitinI27 from Gly-90 to create the TitinI27ΔC domain and then appended variable test sequences at the new C terminus, followed by the ssrA degron tag. To follow the progress of degradation of substrates containing TitinI27ΔC by fluorescence in real time, an mEGFP domain was placed at the N terminus of the TitinI27 domain. Three variants of TitinI27ΔC—WT and single amino acid mutations Y9P and V13P—were used, corresponding to three tiers of domain stability from high to low. This class of substrates is referred to simply as TitinI27ΔC substrates in this study (Fig. 6*A*).

We validated the TitinI27ΔC substrates devoid of test sequences by comparing their degradation kinetics with published results for single TitinI27 domains. The degradation rate of the latter has been well characterized to be dependent on its stability (13, 37). In our TitinI27ΔC substrates, the addition of the N-terminal mEGFP domain does not fundamentally change this trend: WT TitinI27ΔC was the slowest to degrade and V13P the fastest (Fig. 6*B*). The relative ATPase activities of ClpX and ClpXP (Fig. 6*C*) were as anticipated from published data (13, 38). Specifically, in the presence of TitinI27ΔC substrates, the ATP hydrolysis rate of ClpX(P) increased when the stability of the TitinI27ΔC was reduced. However, regarding the ATPase rates of ClpXP, the difference between Y9P and V13P was not statistically significant (Fig. 6*C*). The energy expenditure, calculated as the ratio between ATP hydrolysis rate and the substrate degradation rate, showed clear dependence on TitinI27 stability: WT substrates, which are the most stable, cost significantly more ATP per substrate degraded compared to Y9P and V13P variants (Fig. 6*D*). Therefore, the three variants of our custom Titini27 substrates were processed by ClpX and ClpXP as predicted by the known properties of ClpXP and substrate. We used this validated set of designed substrates to assess the impact of different test sequences on overall degradation rates.

### The effect of substrate tail sequence on substrate degradation rate is dependent on substrate stability

**Figure 7:**
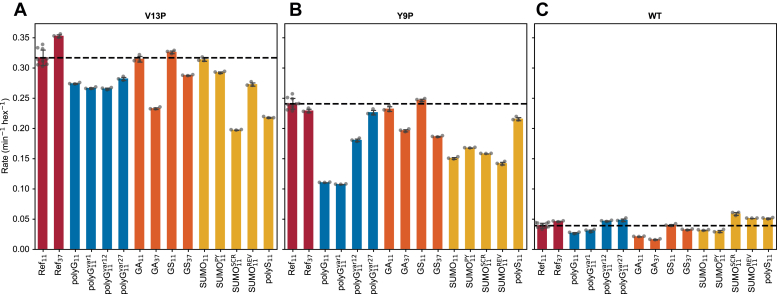
**Degradation of the TitinI27ΔC substrates with diverse tail sequence motifs by ClpXP.***A*, degradation of the V13P variant of the TitinI27ΔC substrates with different tail sequence motifs. Substrate tail motifs were grouped in four categories: Ref (*red bars*), polyG (*blue bars*), GAr and GSr (*orange bars*), SUMO and polyS (*yellow bars*). Ref_11_ has nine biological replicates while the others have three for each tail motif. The average degradation rate for Ref_11_ is indicated as the horizontal dotted line. Degradation rate for Ref_11_ substrate is statistically different from most other substrates in 14 pairwise comparisons between Ref_11_ and 13 other sequence motifs (*p* < 0.05/14 in Bonferroni method), except for GA_11_, GS_11_, and SUMO_11_. *B*, same as (*A*), but with Y9P TitinI27ΔC. Ref_11_ is statistically different from most samples, except for Ref_37_, GA_11_, and GS_11_, using the same statistical criterion as in (*A*). *C*, same as (*A*), but with WT TitinI27ΔC. Ref_11_ is statistically different from all samples except for GS_11_, using the same statistical criterion as in (*A*). GAr, glycine-alanine repeat; GSr, glycine-serine repeat; polyG, polyglycine.

Using the performance of Ref_11_ as the benchmark, we tested the polyG sequences, GAr, GSr and SUMO_11_, and its derivatives using three variants of TitinI27ΔC substrates (Fig. 7). Among polyG_11_ and the derived sequences, the extent to which they decreased the rate of degradation was affected by the stability of the TitinI27ΔC (Fig. 7, *blue bars*). For V13P TitinI27ΔC, which is the least stable, all polyG tails supported similar degradation rates, which were slightly reduced from that of Ref_11_ (Fig. 7*A*, *blue bars*). For Y9P TitinI27ΔC, polyG_11_, and polyG_11_^var1^ had strong inhibitory effects, while polyG_11_^var12^ had a weaker inhibitory effect; polyG_11_^var27^ had no inhibitory effects (Fig. 7*B*, *blue bars*). For WT TitinI27ΔC, only polyG_11_ and polyG_11_^var1^ inhibited substrate degradation (Fig. 7*C*, *blue bars*).

Unlike polyG_11_, the inhibitory effect of GA_11_ appeared only in the WT TitinI27ΔC variant (Fig. 7*C*, *orange bars*). In contrast, GS_11_ did not inhibit TitinI27ΔC among any of the three stability variants (Fig. 7, *A*–*C*, *orange bars*). However, both GAr and GSr exhibited a length-dependent effect. GA_37_ had inhibitory effects on all three Titini27ΔC variants, with the effect on the WT TitinI27ΔC the strongest; GS_37_ had similar broad spectrum effects but the magnitude of inhibition was smaller (Fig. 7, *A*–*C*, *orange bars*).

The pattern of degradation inhibition by SUMO_11_ was similar to that of polyG_11_. For V13P TitinI27ΔC, SUMO_11_, SUMO_11_^PY^, and SUMO_11_^REV^ had little to no effect on its degradation; however, SUMO_11_^SCR^ had a strong inhibitory effect (Fig. 7*A*, *yellow bars*). For Y9P variants, SUMO_11_ and all of its derived sequences showed significant inhibition (Fig. 7*B*, *yellow bars*). For WT TitinI27ΔC, only SUMO_11_ and SUMO_11_^PY^ had inhibitory effects (Fig. 7*C*, *yellow bars*). Interestingly, SUMO_11_^SCR^ and SUMO_11_^REV^ increased degradation rates of WT TitinI27ΔC compared to Ref_11_. In contrast to SUMO11 and its derivatives, polyS_11_ did not show any strong inhibitory effects on Y9P or WT Titini27ΔC variants but surprisingly reduced the degradation rate of the V13P variants (Fig. 7, *A*–*C*, *yellow bars*).

In general, Ref_11_ was among the most effective at supporting fast degradation of substrates. For all slippery sequences identified in the TIRF experiments, the strength of their inhibitory effects relative to Ref_11_ depended on the substrate domain stability. Among three tiers of substrate stabilities, the intermediate stability Y9P TitinI27ΔC most sensitively reflected the effects of the slippery sequences, which were identified in the TIRF assays.

### The effect of substrate tail sequence on ClpX ATPase activity

The ability for ClpX(P) to adapt its ATP hydrolysis rate in response to substrate stability indicates that a stalled substrate might restrict ATPase activity of ClpX(P). This potentially could be achieved by the coupling between mechanical conformational change and ATP hydrolysis, as the movement of the pore-1 loops is restricted. It is unclear whether pore-1 loops have more freedom of movement when engaging a slippery sequence under load. We therefore characterized the impact of substrate tail sequences on ClpX(P) ATP consumption. Because ClpP may also play a role in regulating ClpX ATPase activity, we first characterized the effect of test sequences on ATPase activity of ClpX in the absence of ClpP, using the effect of Ref_11_ as the benchmark.

**Figure 8:**
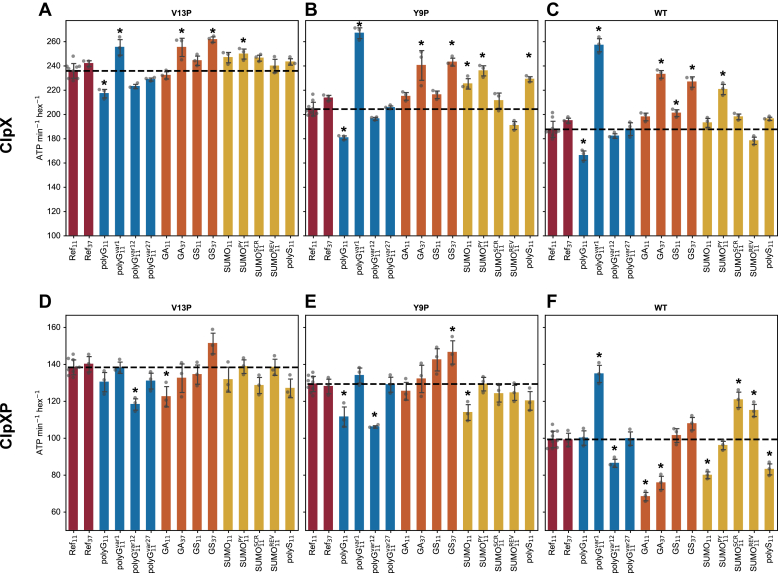
**ATPase activity of ClpX and ClpXP when processing TitinI27ΔC with diverse tail sequence motifs and different substrate domain stabilities.***A–C*, ATPase activity for ClpX when processing TitinI27ΔC of different tail sequences motifs, with V13P (*A*), Y9P (*B*), and WT (*C*) variants. Substrate tail motifs were grouped in four categories, Ref (*red bars*), polyG (*blue bars*), GAr and GSr (*orange bars*), SUMO and polyS (*yellow bars*). Error bars represent SDs. ATPase activity of ClpX when processing Ref_11_ is indicated as the horizontal dotted line across the plots, which is used as the benchmark for statistical comparisons. Conditions that led to differences of statistical significance (*p* < 0.05/14 in Bonferroni method) are marked with asterisks ("∗"). Note that *y*-axis begins at 100 ATP per min per ClpX hexamer, which is close to the basal ClpX ATPase activity in the absence of ssrA-tagged substrate, as shown in Figure 6*C*. *D–F*, ATPase activity for ClpXP when processing TitinI27ΔC of different tail sequences motifs, with V13P (*D*), Y9P (*E*), and WT (*F*) variants. All other conditions and analyses are the same as in (*A–C*). Note that *y*-axis begins at 50 ATP per min per ClpX hexamer, which is close to the basal ClpXP ATPase activity in the absence of ssrA-tagged substrate, as shown in Figure 6*C*. GAr, glycine-alanine repeat; GSr, glycine-serine repeat.

Among polyG motifs (Fig. 8, *A*–*C*, *blue bars*), polyG_11_^var12^ and polyG_11_^var27^ had a similar effect on ClpX ATPase activity as Ref_11_ for all TitinI27ΔC variants. Relative to Ref_11_, ClpX ATPase activity was only marginally lower with polyG_11_. The reduction was on par with the ssrA-only substrates (Fig. 6*B*), which could be caused by a lower ClpX occupancy by polyG_11_ substrates as a result of its higher dissociation rate. However, polyG_11_^var1^ in Y9P and WT TitinI27ΔC led to much higher ClpX ATPase activity than Ref_11_ (Fig. 8, *B* and *C*).

In all TitinI27ΔC variants, the effects of GA_11_ and GS_11_ showed no significant differences from that of Ref_11_ (Fig. 8, *A*–*C*, *orange bars*). However, GA_37_ and GS_37_ led to higher ATP hydrolysis in Y9P and WT TitinI27ΔC compared to Ref_11_ (Fig. 8, *B* and *C*).

SUMO_11_ had effects similar to those of Ref_11_ in V13P and WT TitinI27ΔC (Fig. 8, *A* and *C, yellow bars*) but had slightly higher ClpX ATPase activity compared to Ref_11_ in Y9P TitinI27ΔC (Fig. 8*B*, *yellow bars*). Among SUMO_11_ derivatives, SUMO_11_^PY^ caused higher ClpX ATPase activity than Ref_11_ did in Y9P and WT TitinI27ΔC (Fig. 8, *B* and *C*); the other constructs showed no significance differences from Ref_11_ constructs. The effects of polyS_11_ on ClpX ATPase activity was identical with SUMO_11_ (Fig. 8, *A*–*C*, *yellow bars*)

In summary, ClpX ATPase activity was relatively uniform for most test sequences. However, polyG_11_^var1^, GA_37_, GS_37_, and SUMO_11_^PY^ stood out for higher ClpX ATPase activity in more stable TitinI27ΔC variants when compared with Ref_11_.

### The effect of substrate tail sequence on ClpXP ATPase activity

When ClpP was bound to ClpX, ATP hydrolysis became more uniform for different tail sequences in V13P and Y9P TitinI27ΔC. Notably, for V13P substrates, ClpXP ATPase rates differed statistically only for substrates with polyG_11_^var12^ and GA_11_ tails among all test sequences (Fig. 8*D*).

For polyG sequences, several changes to ClpX ATPase activity occurred in the presence of ClpP. Compared to Ref_11_, polyG_11_ sequence had a lower ClpXP ATPase rate in Y9P TitinI27ΔC (Fig. 8*E* *blue bars*), while polyG_11_^var1^ caused a large increase in ATPase rate in WT TitinI27ΔC (Fig. 8*F* *blue bars*). Surprisingly, polyG_11_^var12^ in all three TitinI27ΔC variants led to lower ATPase activity compared to Ref_11_, and this effect was absent in ClpX (Fig. 8, *D*–*F* *blue bars*). The polyG_11_^var27^ tail behaved similarly to Ref_11_, as was seen with ClpX.

In ClpX, GA_37_ induced higher ATPase activity than Ref_11_ did, but this effect was not observed in ClpXP (Fig. 8, *D*–*F* *orange bars*). GA_11_ and GA_37_ in WT TitinI27ΔC both led to lower ClpXP ATPase activity compared to Ref_11_. (Fig. 8*F*). GS_11_ had similar effects on ClpXP ATPase activity as Ref_11_ for all TitinI27ΔC variants. GS_37_ caused a minor increase of ATP hydrolysis rate in Y9P TitinI27ΔC compared to Ref_11_ (Fig. 8, *D*–*F*).

For SUMO_11_ and related sequences (Fig. 8, *D*–*F*, *yellow bars*), their effects on ClpXP ATPase activity were similar to Ref_11_ in V13P and Y9P TitinI27 variants. In WT TitinI27ΔC, SUMO_11_ and polyS_11_ caused lower ClpXP ATPase activity than Ref_11_ did, while SUMO_11_^SCR^ and SUMO_11_^REV^ raised ClpXP ATPase activity (Fig. 8*F*).

In general, upon ClpP binding, several sequence motifs were associated with lower ClpXP ATPase activity when compared with Ref_11_. These cases can also be grouped in two separate categories. The low ClpXP ATP hydrolysis rate by GA_11_, GA_37_, SUMO_11_, and polyS_11_ was dependent on substrate stability; in contrast, polyG_11_^var12^ was associated with lower ClpXP ATP hydrolysis rate in all substrate stabilities. In WT TitinI27ΔC alone, polyG_11_^var27^, SUMO_11_^SCR^, and SUMO_11_^REV^ were associated with higher ClpXP ATPase activity compared to Ref_11_.

### The effect of substrate tail sequence on ATP cost of substrate degradation

Lowering ClpXP ATPase activity conserves ATP spending per time unit, but a more prolonged unfolding process could increase the cumulative ATP cost per substrate. The multiplicative product of the fuel consumption rate and the time for degradation determines the energy cost per substrate of the degradation process. However, it is not clear whether the balance of ATPase rates and degradation rates is maintained regardless of tail sequence motifs. To investigate this question, we examined the energy efficiency using the ATP costs of degrading Ref_11_ substrates as benchmarks. ATP cost per substrate degradation can be estimated by dividing ATP hydrolysis rate by the corresponding substrate degradation rate.

**Figure 9:**
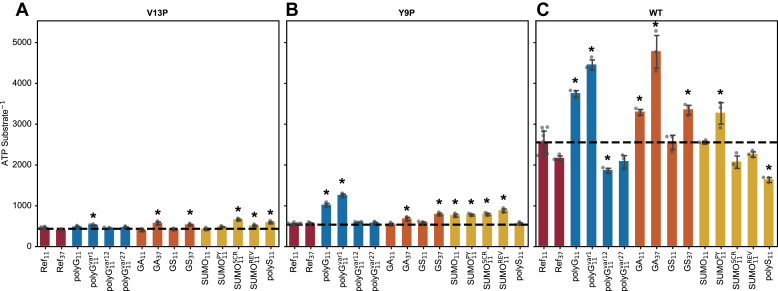
**ATP cost of degradation of TitinI27ΔC with diverse tail sequence motifs and different substrate domain stabilities.***A*, ATP cost of degradation of V13P variant of TitinI27ΔC substrates. Substrate tail motifs are color coded in four categories as in Figures 7 and 8. The value for each construct was calculated by taking the ATPase rate over substrate degradation rate. Sequence motifs that incur statistically different ATP costs (*p* < 0.05/14 in Bonferroni method) compared to Ref_11_ are marked with asterisks ("∗"). *B*, same as (*A*), but measured with Y9P variant of the TitinI27ΔC substrates. *C*, same as (*A*), but measured with WT variant of the TItinI27ΔC substrates.

For polyG sequences (Fig. 9 *blue bars*), polyG_11_ and polyG_11_^var1^ dramatically increased ATP costs in Y9P and WT TitinI27ΔC, with the latter incurring higher ATP cost. PolyG_11_^var12^ and polyG_11_^var27^ had similar effects on ATP cost as Ref_11_ in all three TitinI27ΔC variants.

ATP cost was increased by GA_11_ only in WT TitinI27ΔC (Fig. 9*C* *orange bars*). TitinI27ΔC substrates with GS_11_ tails were degraded at similar efficiency as those with Ref_11_ (Fig. 9, *A*–*C* *orange bars*). GA37 and GS_37_ induced higher ATP cost in all three TitinI27ΔC variants, but the extent of increase differs depending on domain stability (Fig. 9, *A*–*C* *orange bars*).

For SUMO_11_ and related sequences, their effects on ATP cost also depended on domain stability (Fig. 9, *A*–*C* *yellow bars*). For V13P TitinI27ΔC, ATP cost was increased by SUMO_11_^SCR^ and SUMO_11_^REV^ (Fig. 9*A*). For Y9P TitinI27ΔC, all SUMO_11_ and its derived sequences increased the substrate ATP cost (Fig. 9*B*). For WT TitinI27ΔC, only SUMO_11_^PY^ increased ATP cost while other sequences had similar effect as Ref_11_ (Fig. 9*C*). Unlike SUMO_11_, polyS_11_ allowed efficient degradation of WT and Y9P TitinI27ΔC substrates but led to higher energy cost for V13P TitinI27ΔC (Fig. 9, *A*–*C*).

In summary, ATP cost for substrate degradation was strongly impacted by domain stability as well as the tail sequences. The effect of tail sequence on ATP cost was dependent on domain stability, and the effect became more prominent with highly stable substrates. For stable substrates, although polyG and GAr had significantly different effects on ClpXP ATPase activity, both incurred higher ATP cost than did Ref_11_.

## Discussion

ClpXP, like other AAA+ proteases, such as the 26S proteasome, is capable of unfolding and degrading protein substrates with a wide range of stabilities and amino acid compositions, despite its relatively simple architecture. Importantly, processive substrate degradation by AAA+ proteases demands efficiencies in both unfolding and retention of the substrate. Crucial to substrate unfolding is directional substrate translocation through the central pore of the AAA+ protease, which is dependent on the interaction between the conserved aromatic pore loops and the substrate polypeptide. In this model, rate and efficiency of substrate unfolding depend on whether the force of power strokes from the protease can be delivered *via* these pore loop interactions to the substrate to overcome its mechanical stability. It has been shown that the amino acid composition of the substrate can strongly affect force delivery (19). However, it is not exactly clear whether changes in the quality of pore loop interactions with the substrate can also affect the processivity of substrate degradation. In principle, adventitious substrate escapes due to reduced unfolding efficiency can have significant effects on the kinetics of protein degradation. Therefore, such changes in substrate retention may strongly influence protein turnover in cells. In this study, we focused on whether sequence motifs known to reduce substrate degradation rates may concomitantly lead to increased substrate dissociation.

To study the general question of substrate retention by AAA+ proteases, we used ClpX as a model. Hypothetically, substrate retention can be passively mediated by substrate affinity to ClpX but also by activities of ClpX, which may dynamically accommodate low affinity substrates. Factors independent of pore-1 loops might also affect substrate retention. The scope of these complex interactions between factors contributing to substrate degradation has not been fully explored. Here, we have designed a single molecule assay and showed that substrate slippage is strongly influenced by the composition of the substrate tail sequence.

A critical aspect of the experimental design relied on an established model of substrate slippage. Specifically, it has been observed that substrate slippage from the proteasome requires the juxtaposition of a stably folded domain and a simple sequence (20, 21, 22). Thus, while we designed the TIRF assay using the MTX-stabilized DHFR for its utility in simplifying the kinetic path by excluding successful unfolding, the design also has the benefit of promoting slippage. This effect is further supported by recent single molecule measurements on 26S proteasomes using folded TitinI27 domains of varied stabilities; slippages are found to be more frequent when the proteasome engages the most stable form of TitinI27 (39).

### Slippage rate and slippage distance both regulate substrate dissociation

Our initial TIRF measurements show that substrate dissociation is a combined outcome of multiple factors. One such factor is substrate tail length (Fig. 2*G*). This finding can be readily rationalized by the well-established slippage distance model inferred from optical trap studies (9, 14). In this model, substrate slippage occurs after a loss of interaction between the pore-1 loops and the substrate, which allows the substrate to be pulled back freely by the opposing force applied by the laser trap. In optical trap experiments, the backtracking tends to end at a distance centered on 30 to 40 aa, implying a recovery of pore loop engagement after transient disengagement of a fixed time period. For a substrate with a fixed slippage frequency and a fixed probability of dissociation incurred by each slippage event, the dwell time distribution of the fully engaged substrate can be modeled using single-exponential decay, which fits well to our TIRF data. However, because substrate association to ClpX is a multistep process (40), it is likely that the reverse process is equally complex. During substrate dissociation, fast kinetic steps under 1 s have been reported from stopped-flow experiments (40), but this is beyond the temporal resolution of our TIRF system. Nonetheless, the single kinetic parameter, τ, was sufficient for distinguishing the diverse effects of substrate sequences on substrate retention in our study.

The simplest slippage distance model predicts that substrate retention time should increase exponentially with extended tail length. This was not exactly observed in our TIRF experiments: the trend of substrate dwell time increase does not suggest an exponential increase. This result could be due to the limitations of the TIRF method; for example, binding events with very long dwell times may be subject to increased risk of photobleaching. Alternatively, the result could reflect a biochemical property of ClpX, whereby the unfoldase can overcome stalling by unique mechanisms. This uncertainty highlights the fact that the single exponential decay model is an approximation to a complex process and that our TIRF method has a specific range of temporal resolution. In our study, we focused on shorter tail lengths, wherein TIRF experiments and published optical trap experiments agree best (9, 14). In these cases, progressively shortening tail length would allow dissociation rate to approach the slippage rate, according to the slippage distance model. Therefore, shorter sequences increase the sensitivity of the TIRF assay for differentiating slippery motifs from nonslippery ones.

### The relationship between substrate slippage and the ATPase cycle

In the single molecule experiments, while substrate dissociation reflects a loss of grip by ClpX, the exact cause for the slippage is unclear. One reason for the uncertainty is the stochastic nature of the ATPase duty cycle of ClpX. The cycle consists of short bursts of power strokes, which are separated by long dwell phases (11). Hypothetically, during bursts, when power strokes are delivered, slippage might occur if the substrate polypeptide resists the sudden pulling motion of the pore loop, which may break the grip by ClpX. It is also likely that extended dwell phases of the ATPase might contribute to slippages due to prolonged influences of stochastic motions. Moreover, the ClpX ATPase cycle appears to be sensitive to substrate stability and is heavily regulated by ClpP. Knowing the relative contributions of bursts and dwells to substrate slippage would help elucidate the exact mechanism by which a substrate fails to be degraded. One drawback of our current TIRF system is the inability to simultaneously control and monitor the ATPase activity of ClpX. However, with the help of new high resolution cryo-EM structures, FRET reporters for ClpX ATPase cycle could be developed.

### Substrate dissociation conserves energies for ClpXP for slippery substrates

It has been proposed that a polyG tract inhibits degradation by allowing pore-1 loops to slip along the gripped tract, thus failing to gain traction for pulling the substrate, while causing ClpX to perform futile cycles of ATP hydrolysis (19, 25). Our present results offer a more nuanced view into the cause of inhibition. In the TIRF experiments using polyG_11_ (Figs. 3 and 4), we demonstrate that a polyG tract is, as was expected, slippery. We also show that the slipperiness of the polyG_11_ is dependent on the position of the motif within the tail. The slipperiness of polyG_11_ is maximized only when it is adjacent to the folded domain. Recent cryo-EM structures of ClpXP show that at preunfolding dwell stage, the 10 to 12 amino acid unstructured region next to the folded domain of the substrate sits within the narrowest stretch of the central pore, gripped by the pore-1 loops (5, 6). Therefore, these results imply that a polyG_11_ sequence is badly retained by pore-1 loops.

The result for polyG_11_ dwell times on ClpX correlates well with the TitinI27ΔC degradation experiments with ClpXP. In both cases, the dwell time and the degradation rate are affected by the position of the polyG_11_ within the tail (Fig. 7). We also find that the inhibitory effect of polyG_11_ was dependent on substrate stability, reinforcing the importance of the resistant domain as a cause of degradation inhibition. However, while polyG_11_ induced significantly higher ATP cost per substrate degraded, this was not caused by a dramatically higher rate of ATP hydrolysis per time per unit of ClpXP when compared with the effects of Ref_11_. In other words, in the case of polyG_11_, ATPase cycles are more futile but not faster. In comparison, with polyG_11_^var1^, the ATPase rate became much higher, contributing to even greater ATP cost per substrate degraded compared to polyG_11_. While polyG_11_^var1^ supported a better retention rate with ClpX than did polyG_11_, this did not translate into a better unfolding rate. Instead, the higher dissociation rate for polyG_11_ substrates may have allowed ClpXP to preserve ATP in its unengaged resting state. Conversely, the more frequent recovery of slippages by polyG_11_^var1^ forces ClpXP to rapidly translocate the substrate back to the preunfolding dwell stage causing massive waste of ATP. This model agrees with the proposal that, in effect, ClpXP inclines to release stable substrates to prevent sequestration of the enzyme, while preferentially processing substrates of lower stability (41).

We also noted that ClpXP ATPase activity is consistently lower when degrading substrates with polyG_11_^var12^. PolyG_11_^var12^ was retained well by ClpX, and the ATP cost for degrading polyG_11_^var12^ substrates is similar to Ref_11_ and Ref_37_. Based on the cryo-EM structures, the polyG_11_ motif in polyG_11_^var12^ should be below the spiral staircase of pore-1 loops during substrate unfolding. These results indicate that the polyG_11_ motif of polyG_11_^var12^ does not interfere with the efficiency of unfolding. Instead, it reduces the ATPase activity through an unknown mechanism, which is activated in ClpXP but not ClpX.

### GAr sequences may lock ClpX conformation by chemomechanical coupling

Sequences similar to GAr are found in a number of proteins in diseases that involve disrupted protein degradation, such as the Epstein-Barr virus nuclear antigen-1, and the poly-GA aggregates expressed from c9orf72 gene (33, 42, 43). GAr sequences are often compared with glycine-rich sequences, partly because of its low sequence complexity and the rich presence of glycine residues. Past studies using ensemble assays in ClpXP have shown that GAr may inhibit the degradation of DHFR and WT TitinI27 but not of GFP (19, 24). In the case of degradation inhibition, GAr could reduce forward degradation without changing dissociation rate, but the study did not consider the retentive effect of long tail length, making it difficult to conclude whether GAr is truly a slippery sequence or not (24, 25).

Our dwell time experiments showed that unlike polyG, the GA_11_ sequence is not slippery (Fig. 4). Instead, the hydrophobicity of the alanine played an important role, as the similarly constructed substrates with GSr tails had a much higher dissociation rate compared to GAr. These findings were consistent with the evidence that when degrading GFP, ClpXP has a stronger grip over hydrophobic residues than polar and charged residues (19). Consequently, the inhibitory effect of GAr is not explained by its slipperiness.

In the bulk solution degradation assays, we recapitulated the finding that the WT TitinI27ΔC degradation was reduced by GA_11_. However, this effect was mitigated by lowering TitinI27ΔC stability. This result implies that the reason GAr selectively inhibits substrate degradation could be related to differences in the energy barrier for substrate unfolding. An analysis of the ATP hydrolysis rate revealed that ClpXP experienced a sharp reduction in ATPase activity when degrading WT TitinI27ΔC with GA_11_ tail when compared with Ref_11_ (Fig. 8). This points to the interdependence between substrate unfolding and ClpXP ATPase rate as another cause for degradation inhibition.

Evidence for this chemomechanical coupling is manifold. In single molecule optical traps, the power strokes of ClpXP can be severely limited by either ATPγS binding or point mutations in the ATP-binding sites (11, 12). Conversely, the ATPase rate of ClpXP can be limited by increasing the bulk of the pore-1 loop, which mimics the restriction of pore-1 loop movements by the engaged substrate (44). Therefore, one hypothesis for the effect of GA_11_ is that the tightly engaged substrate may impede the movement of the pore loops, thus restricting the ATPase cycle of ClpXP. This impediment to pore loops requires a stable substrate that resists translocation. Importantly, this inhibition is also dependent on ClpP. This implies an underlying allosteric regulation of ClpX activity upon ClpP binding, the mechanism of which is unclear.

The inhibition by GAr also showed a length-dependent effect independent of substrate stability. GA_37_ had an impact on degradation of all three variants of TitinI27ΔC substrates, unlike GA_11_, which only impacted degradation of WT TitinI27ΔC (Fig. 7). In TIRF experiments, GA_37_ led to a much higher substrate dwell time than Ref_37_ (Fig. 4); in comparison, GA_11_ and Ref_11_ have similar values of *τ*. The length-dependent effect was also observed in GSr, which was more slippery compared to GAr. Therefore, this effect could be unrelated to the grip by pore-1 loops. While there are many potential causes, one attractive hypothesis is that the combination of length and simple repeats of amino acids leads to secondary structures that require extra energy or time to translocate. It was shown in single molecule FRET studies with proteasomes that serine-rich sequences are poor degradation initiation sites for proteasomal substrates due to a slow rate of insertion into the central pore (45). This observation suggests that GSr sequences might have a lesser degree of freedom. Likewise, long stretches of GAr have also been associated with structured aggregates (46). Unusual structural elements in the substrate tail might pose additional challenges for the unfoldase, potentially by adding another energy barrier before substrate degradation.

The tendency for the unfoldase to retain the GAr sequence in difficult-to-unfold substrates could have important physiological consequences in cells. This class of substrate might choke the unfoldase and deplete its cellular pool, thus reducing the total proteolytic capacity of the cell. It has been shown with cryo-EM tomography in cells expressing poly-GA repeats from C9orf72 gene that 26S proteasomes are recruited to the poly-GA aggregates (47). These proteasomes are enriched in the substrate processing state, implying that the 26S proteasome might be trapped by the aggregates.

### Tail motifs with polar residues

The short dwell time of SUMO_11_ with ClpX indicates an increase in slippage rate compared to Ref_11_ (Fig. 5). Guided by the findings from an independent study (19), we modified the SUMO_11_ to explore potential strategies to rescue the slipperiness of the sequence. However, none of the modifications or shuffling of the sequence we tested fundamentally changed the short dwell time in the TIRF assay. These results indicate that the sequence composition matters more than the positioning of specific amino acids in SUMO_11_.

Interestingly, while the SUMO_11_ have similar values of τ as that of the polyG_11_, the overall degradation rates for TitinI27ΔC substrates with SUMO_11_ sequences tend to be higher than that of polyG_11_. SUMO_11_ also has better energy efficiency than polyG_11_. Given the dissimilarity between the amino acid sequences of the two motifs, it is unlikely that the slipperiness of SUMO_11_ and polyG_11_ share the same mechanism. One among many potential hypotheses is that force transduction from pore-1 loops to the substrate can be achieved not only by high affinity with substrate sidechains but also by steric frictions. In the latter case, a low affinity sequence might still transiently produce sufficient grip. This could explain why polyS_11_, which contains serine residues that have worse affinity with ClpX than alanine residues, still supports efficient protein degradation for stable substrates.

### A general model for degradation inhibition by slippery sequences

Across the evolutionary tree, bulky aromatic residues within pore loops are a common feature of AAA+ proteases. This implies that the underlying biophysical mechanism governing the interaction between the unfoldase and the protein substrate is conserved. Therefore, the substrate sequence preference of ClpX revealed in this study has the potential to be applied to a wide range of AAA+ ATPases. For example, in many aspects, our work is in agreement with findings in eukaryotic proteasomes. Specifically, our results using TitinI27ΔC substrates uphold the classic model that proteasomal degradation can be frustrated by the cooperation of a stable substrate domain and a well-spaced unstructured motif (20, 21, 42). It has been proposed that a common feature of these unstructured motifs is their low complexity (20, 23). However, the crude complexity assessment takes little account of amino acid properties, but does award high scores to compositional diversity. The TIFR microscopy results for polyG, GAr, and GSr show that amino acid sequences interact with ClpX in diverse ways, despite their similarly low computational complexity, suggesting that the mechanisms of inhibition by LCRs are not uniform. On the other hand, the results from SUMO_11_, which is more complex than GAr and GSr, yet also more inhibitory under certain conditions, highlights the shortcomings of LCR-based predictions of degradation (a plot of LCR value *versus τ* is shown in Fig. S9). Our TIRF microscopy results show that the specific features of the unstructured motifs matter. In this view, the parameter of sequence complexity is less relevant than hydrophobicity of the sequence.

This observation leads to an updated model for understanding the factors that affect slippage frequency. In this model, substrate stability determines the threshold force required to unfold; the sequence of the unstructured motif determines whether grip can be maintained when the AAA+ ATPase delivers the requisite unfolding force. Thus, the interplay of these two factors may create many combinations of domain stabilities and sequence motifs that could result in increased slippage frequency. Whether a loss of grip can lead to substrate dissociation in turn depends on substrate tail length and other unknown factors. The presence of multiple interacting and tunable factors in our model suggests that substrate degradation rate can be broadly tuned without any modifications to the degrons. This is in agreement with the finding that different substrate tail sequence motifs could tune steady-state protein levels across a wide dynamic range in proteasome-mediated degradation (48).

Importantly, this simple model permits complex sequences to inhibit degradation for a coevolved substrate domain. For example, partial processing of a substrate by AAA+ unfoldase is used by cells as a regulatory mechanism. Thus, transcription factors NF-κB and Gli3 have been found to be activated by partial degradation by the proteasome (49, 50). In both examples, processing interruption and activation has been shown to require the combination of a folded domain and a stop signal adjacent to the folded domain, implying the association with the grip by pore-1 loops (51). However, while the glycine-rich region of the P105 is easily identifiable due to its low complexity, the stop signal of the Gli3 is less apparent; it is rich with serine, proline, arginine, and glutamine residues, similar to SUMO_11_. The example of SUMO_11_ shows that there might be overlooked motifs of higher complexity, working in combination with a moderately stable substrate domain, to help substrates evade AAA+ protease degradation.

Finally, the presence of substrates that are ineffectively degraded can have multiple impacts on cellular physiology. For example, a number of neurodegenerative diseases are associated with expression and accumulation of proteins with unusual sequence features (52, 53, 54). While it is still unclear whether changes in proteasomal activity contributes to the pathology of these diseases, the data reported here shed light on one aspect of degradation inefficiency—substrate escape—and its relationship to the amino acid composition of tracts engaging the translocation apparatus.

## Experimental procedures

### Plasmids

The plasmid encoding ClpX-ΔN pseudohexamer with C-terminal avitag (ClpX6B) and C-terminally tagged ClpP monomers were derived from the two plasmids respectively from pACYC-Duet-1-ClpX6-ΔN (Addgene # 71147) kindly provided by Dr T. Baker (MIT) and from ClpP with N-terminal His-tag (pCPX01) gifted by Dr H. Nakai (Georgetown University Medical Center). For making cpGFP::DHFR::test-sequence::ssrA constructs, the cp7-140-sfGFP was a gift from Dr G. S. Waldo, which was then cloned into a pET028a(+) vector with an N-terminal 6his-tag and C-terminal ssrA tag. Three cysteine residues were introduced into the N-terminal unstructured region of the cpGFP by site-directed mutagenesis, spaced at least 8 amino acids apart to allow subsequent labeling by thiol-reactive fluorophores; the separation of cysteine residues was intended to reduce self-quenching of fluorophores (thus the N terminus sequence reads MGCSSHHHHHHHSCSGL-VPRGSCHMGGTS). Site-directed mutagenesis was conducted according to the manufacturer's protocol of Agilent QuikChange II kit. The *E. coli* DHFR domain was obtained by PCR amplification from pET15b His6-ecDHFR (WT), which was a gift from a gift from Dr T. Wandless (Addgene plasmid # 73188), and was then inserted between the cpGFP and ssrA using the NEBuilder kit. A HindIII site was added between the DHFR and ssrA tag using site-directed mutagenesis. The HindIII site allowed for insertion of synthesized dsDNA cassettes expressing different test sequences using NEBuilder Hi-Fi DNA assembly kit (New England BioLabs). A similar strategy was used to construct the 6his-mEGFP-TitinI27ΔC-test-sequence-ssrA. An intermediate construct, 6his-mEGFP-TitinI27-BamHI-ssrA, was first created by inserting a synthesized dsDNA fragment of full length TitinI27 domain, containing a 3′ BamHI site, into an 6his-mEGFP-ssrA gene in pET028a(+) vector using NEBuilder Hi-Fi DNA assembly kit, between the mEGFP domain and ssrA tag. The TitinI27ΔC was made by utilizing the native BsaI site within TitinI27 in combination with the BamHI site when inserting synthesized test sequence dsDNA using NEBuilder Hi-F- DNA assembly kit. Y9P and V13P mutations of TitinI27 domains were made by PCR site-directed mutagenesis.

### Protein expression, purification, and labeling

All proteins were expressed in *E. coli* BLR(DE3) strain. For expressing ClpX pseudohexamer, the ClpX6B was coexpressed with biotin ligase (BirA), as previously described (24), in terrific broth media, and incubated with 50 μM biotin and 0.8 mM IPTG at 16 °C overnight. For ClpP and mEGFP-TItinI27 substrates, expressions were induced by 1 mM IPTG and incubated for 3 h at 37 °C. For cpGFP-DHFR substrates, expressions were induced by 1 mM IPTG and incubated at 16 °C overnight. Harvested cells were frozen at −80 °C before lysis.

Pelleted cells were processed using BugBuster Protein Extraction Reagent (EMD Millipore) according to the manufacturer protocol. Briefly, the proprietary detergent-based lysis buffer was spiked with rLysozyme (EMD Millipore) at 5 KU per g cell pellet, Benzonase (EMD Millipore) at 125 U per g cell pellet, 1 mM DTT, and EDTA-free cOmplete protease cocktail (Roche). Cells were lysed by mixing with the completed lysis buffer and incubated at room temperature (RT) for 20 min for substrates or 1 h at 4 °C for ClpX6B.

ClpX6B was affinity purified using Pierce monomeric Avidin agarose (ThermoFisher) in a packed gravity flow column. After binding, the column was washed with a PBS buffer (100 mM PBS, pH 7, 150 mM NaCl, 10% glycerol) and eluted with the same PBS buffer supplemented with 2 mM D-biotin. ClpP and all substrates were purified using Ni-NTA agarose with gravity columns. The protein-bound column was washed with a PBS wash buffer (50 mM PBS, pH 8, 300 mM NaCl, 1 mM DTT, 20 mM imidazole) and eluted with the same buffer supplemented with 250 mM imidazole. ClpX6B was buffer exchanged into Hepes storage buffer (25 mM Hepes, pH 7.4, 100 mM KCl, 10%l glycerol) by first concentrating the elution fractions using an Amicon centrifugation filter to about 1/10 of the original volume (10K MWCO, EMD Millipore), then dialyzed overnight in the Hepes storage buffer using a slide-A-lyzer cassette (Pierce) with 3K MWCO. For ClpP and mEGFP-TitinI27 substrates, elution fractions were processed by gel filtration using Sephadex G-25 in PD-10 desalting columns (GE Healthcare/Cytiva) equilibrated with Hepes storage buffer. The cpGFP-DHFR substrates were exchanged into labeling buffer (25 mM Hepes, pH 7.0, 150 mM KCl, 10% glycerol, 1 mM DTT) using the same gel filtration method, where the DTT was removed right before the labeling reaction using 0.5 ml Zeba spin desalting columns (ThermoFisher). Proteins were quantified using Bradford assay (Pierce, 23200) using BSA as standard.

The cpGFP-DHFR substrates were labeled using different thiol-reactive maleimide dyes under the same labeling condition. Unless stated otherwise, the ssrA-tagged substrates were labeled with Cy3, while ssrADD mutants were labeled with Cy5. Sulfo-Cy3 or Sulfo-Cy5 (Lumiphore) were kept at around 10:1 M ratio in excess to ∼50 μM of protein, and reactions were conducted at room temperature for 2 h in a vacuum desiccator. In experiments described in supporting information, cpGFP substrates used for optimization of the TIRF system were labeled with alternative thiol-reactive dyes under the same condition and labeling stoichiometry. For ClpX6B, 1 μM of hexamer was labeled with 10 μM Sulfo-Cy5 dye molecules at room temperature for 1 h in a vacuum desiccator. Labeling reactions were stopped by 20 min incubation at room temperature with 5 mM 2-mercaptoethanol. Excess dyes were then removed by 0.5 ml Zeba spin desalting columns, followed by concentrating in Amicon Ultra-0.5 centrifugation filter (EMD Millipore).

### Degradation assay *in vitro*

Fluorescence-based degradation assay was conducted in the ClpX reaction buffer (25 mM Hepes with pH at 7.4, 100 mM KCl, 20 mM MgCl_2_, 10% glycerol). ATP was regenerated using 16 mM creatine phosphate and 3.6 U/ml creatine phosphokinase. Reactions were loaded in a Corning 96-well half-area black flat bottom polystyrene plate with nonbinding surface, and fluorescence were measured using Tecan Spark 20 plate reader by excitation at 485 nm and emission at 535 nm. A photobleaching control for each fluorescent substrate was included in the same measurement to correct for photobleaching caused by repeated readout flashes from the plate reader. Fluorescence was normalized by calculating the fraction between fluorescence at time point t over fluorescence at time 0 (*F*_*t*_*/F*_*0*_). In the bleaching control, the fraction of GFP that remains fluorescent at each time point was calculated using (*F*_*t*_^*Bleach-Ctrl*^*/F*_*0*_^*Bleach-Ctrl*^). The normalized fluorescence of the sample was then corrected for photobleaching by dividing the normalized level with (*F*_*t*_^*Bleach-Ctrl*^*/F*_*0*_^*Bleach-Ctrl*^) ratio for each corresponding time point. The normalized trace was curve fitted with a simple linear model *F(t) = -k∗t + B*, where k is the rate of reduction in fraction of fluorescence.

### ATPase assay *in vitro*

ATPase activity was measured using an NADH-coupled assay, with 1 mM NADH, 2.5 mM phosphoenolpyruvate, and 1/20 diluted pyruvate kinase/lactic dehydrogenase enzymes stock (PK/LDH, where PK is 600–1000 U/ml and LDH at 900–1400 U/ml, Sigma–Aldrich, P0294). Reactions were carried out in the ClpX reaction buffer and were loaded in Corning clear flat bottom half-area 96-well microplates. Reduction of NADH was recorded using Tecan Spark 20 plate reader by measuring absorbance at 340 nm. The background absorbance of the mEGFP as well as the 96-well plate was negligible. The pathlength of the reaction was determined by first measuring the absorbance of a NADH standard from 0 to 1.2 mM, at the same volume as the tested samples in the same type of microplate and then fitting the absorbance-concentration curve by *Abs340 = 6.22 (mM*^*-1*^*cm*^*-1*^*) lightpath (cm) ∗ NADH*. ATP hydrolysis rate was then derived by fitting the reduction of Abs340 over time by *Abs340 = k∗t + c*, where k is equivalent to *ΔAbs340/Δt*, which is then converted to rate by the formula *ΔAbs340/(6.22 ∗ lightpath)*. The background NADH consumption caused by leaky activity of the PK/LDH mix was also measured and subtracted from the results.

### TIRF imaging

Flow cell entry and exit ports were drilled into the standard microscopy slides using a Dremel rotary tool with a 1/16 inch diamond drill bit. Cutouts of channels were made on two layers of Parafilm, which was about 0.26 mm thick according to manufacturer specification (55). The channel cutout was sandwiched between passivated slides and coverslips with the ports aligned, and the assembly was heated on a 70 °C hot plate for 1 min. The entry and exit ports for each channel were 10 mm apart and the channel was about 3 to 4 mm wide, 12 mm long. Thus, each channel could hold about 10 μl volume.

Before the assembly of the flow cell, the drilled slides and coverslips were passivated using DDS-Tween-20 method adapted from the method developed by Hua *et al*. (28). Briefly, slides and coverslips were cleaned in 1% Alconox solution, then by argon plasma for 3 min, then by a base bath of 5% KOH in isopropanol for 3 to 4 h. The slides were then rinsed by DI H_2_O and air dried. The cleaned class was then washed twice with hexane and incubated with 0.3% DDS dissolved in hexane for 2.5 h at room temperature and protected from light. Excess DDS was then washed off with two more hexane washes post-reaction and one wash by MilliQ water.

All steps in the TIRF experiments were performed in the same ClpX reaction buffer used in biochemical assays. First, 25 μl of 0.2 mg/ml biotinylated BSA was flowed into an empty channel and incubated for 5 min. Then, 100 μl of 0.2% Tween-20 was flown in and incubated for 10 min. The channel was then washed with 50 μl of 0.01% Tween-20. Then, 25 μl of 0.2 mg/ml streptavidin in 0.01% Tween-20 was flowed in the channel for 1 min incubation. Excess streptavidin was washed away by 50 μl of ClpX reaction buffer. The channel was then equilibrated with 50 μl of ATP wash buffer (ClpX reaction buffer containing 2 mM ATP). Fifty microliters of 10 nM ClpX preincubated with 2 mM ATP was then flowed in the channel and incubated for 1 min, followed by a 50 μl wash with the ATP wash buffer. Finally, 20 μl of substrate sample mixture that were preheated to 30 °C were flowed in, and the entry and exit ports of the flow cell were plugged by Parafilm to slow evaporation. The substrate sample mix contains 25 μg/ml BSA (Sigma–Aldrich A7030, fat free and protease free), 100 μM MTX, 2 mM Trolox (Millipore Sigma Calbiochem 648471), 5 nM of ssrA and ssrADD substrates, and 2 mM ATP. An oxygen scavenger system is also included in the substrate sample mix, consisting of 16 μg/ml of glucose oxidase (VWR Life Science 0243-100KU), 375 μg/ml catalase (Affymetrix 12885) and 1% D-glucose.

TIRF microscopy was conducted using a modified Olympus IX-81 with an UApoN 100x/1.49 numerical aperture objective, 561 nm and 647 nm laser, and TRF89901 quad band set (Chroma) for Cy3 and Cy5 imaging. The critical angle was approximately 61.2° and the TIR angle was approximately between 64.65° and 68.60°, yielding a spatial constant for decay to 1/3 of the evanescent field between 124 and 88 nm. For imaging with Cy3, the power output of the 561 nm laser was set to 5 mW when using 2 s/frame interval or 2.5 mW when using 1 s/frame interval; for imaging with Cy5, the 647 nm laser was set to 10 mW. The shorter interval was used when the average dwell time of the protein was initially measured to be below 8 s. Images were captured with 100 ms exposure using a Hamamatsu Flash 4.0 camera. TIRF was achieved using an azimuthal method with a steerable mirror set up to minimize interference patterns, as previously described (56). The measurements were carried out at 30 °C. Due to the presence of the acidifying oxygen scavenger system, imaging sessions were kept under 90 min.

### TIRF data analysis

A flow chart for the data processing method is shown in the Fig. S4. Data were processed using a custom built Python script. Each frame of a time course movie was scanned for circular blobs using the Laplacian of Gaussian (LoG) method from a published library package (57). The collected dots were then fitted with a 2D Gaussian function and assessed for its brightness and shape; dots of too high brightness or not circular (defined by extreme ratios of its x and y widths) were filtered out at this step. The remaining dots were then tracked over frames to be compiled into traces for each punctum. The tracking method allowed a 3-frame gap in each trace to account for problems like fluorophore blinking or overly aggressive filtering in previous processing steps. New traces that started within 1 μm of another trace were filtered out. Traces that start on the first frame or end on the last frame of the time course movie were also filtered out due to uncertainties of the lifetime of these puncta. Traces that occupy the same x-y coordinates but at different time points were filtered out due to uncertainties of the uniqueness of the binding events. Traces that start and end on the same frame were also filtered out because of the likelihood of nonspecific interactions or fluorescence impurities. The outputs, which contain the dwell time of individual puncta from multiple time course movies during a single experiment, were pooled into a single database. For each experiment, traces with the same dwell time were counted from the pooled trace databases, and the tallies were sorted for curve-fitting. Curve fittings were done in MATLAB. Dwell times under 120 s were compiled into an empirical cumulative distribution curve using the ecdf function of MATLAB and were fitted using the cdf of exponential distribution *1 – e∧(–t/τ) + c*, where c is the error term limited under ±0.025. Curves were fitted with the least absolute residual robustness mode turned on.

## Data availability

All data are contained in the article and supporting information. Original imaging files are available upon request. The data processing script used to obtain puncta dwell times from original imaging files are deposited at GitHub (https://doi.org/10.5281/zenodo.6494044)

## Supporting information

This article contains supporting information.

## Conflict of interest

The authors declare that they have no conflicts of interest with the contents of this article.

## References

[1] Pohl C., Dikic I. (2019). Cellular quality control by the ubiquitin-proteasome system and autophagy. Science.

[2] Baker T.A., Sauer R.T. (2012). ClpXP, an ATP-powered unfolding and protein-degradation machine. Biochim. Biophys. Acta.

[3] Martin A., Baker T.A., Sauer R.T. (2008). Diverse pore loops of the AAA+ ClpX machine mediate unassisted and adaptor-dependent recognition of ssrA-tagged substrates. Mol. Cell.

[4] Glynn S.E., Martin A., Nager A.R., Baker T.A., Sauer R.T. (2009). Structures of asymmetric ClpX hexamers reveal nucleotide-dependent motions in a AAA+ protein-unfolding machine. Cell.

[5] Fei X., Bell T.A., Jenni S., Stinson B.M., Baker T.A., Harrison S.C. (2020). Structures of the ATP-fueled ClpXP proteolytic machine bound to protein substrate. eLife.

[6] Ripstein Z.A., Vahidi S., Houry W.A., Rubinstein J.L., Kay L.E. (2020). A processive rotary mechanism couples substrate unfolding and proteolysis in the ClpXP degradation machinery. eLife.

[7] Gatsogiannis C., Balogh D., Merino F., Sieber S.A., Raunser S. (2019). Cryo-EM structure of the ClpXP protein degradation machinery. Nat. Struct. Mol. Biol..

[8] Aubin-Tam M.-E., Olivares A.O., Sauer R.T., Baker T.A., Lang M.J. (2011). Single-molecule protein unfolding and translocation by an ATP-fueled proteolytic machine. Cell.

[9] Maillard R.A., Chistol G., Sen M., Righini M., Tan J., Kaiser C.M. (2011). ClpX(P) generates mechanical force to unfold and translocate its protein substrates. Cell.

[10] Shin Y., Davis J.H., Brau R.R., Martin A., Kenniston J.A., Baker T.A. (2009). Single-molecule denaturation and degradation of proteins by the AAA+ ClpXP protease. Proc. Natl. Acad. Sci. U. S. A..

[11] Sen M., Maillard R.A., Nyquist K., Rodriguez-Aliaga P., Pressé S., Martin A. (2013). The ClpXP protease unfolds substrates using a constant rate of pulling but different gears. Cell.

[12] Cordova J.C., Olivares A.O., Shin Y., Stinson B.M., Calmat S., Schmitz K.R. (2014). Stochastic but highly coordinated protein unfolding and translocation by the ClpXP proteolytic machine. Cell.

[13] Kenniston J.A., Baker T.A., Fernandez J.M., Sauer R.T. (2003). Linkage between ATP consumption and mechanical unfolding during the protein processing reactions of an AAA+ degradation machine. Cell.

[14] Iosefson O., Olivares A.O., Baker T.A., Sauer R.T. (2015). Dissection of axial-pore loop function during unfolding and translocation by a AAA+ proteolytic machine. Cell Rep..

[15] Martin A., Baker T.A., Sauer R.T. (2008). Protein unfolding by a AAA+ protease is dependent on ATP-hydrolysis rates and substrate energy landscapes. Nat. Struct. Mol. Biol..

[16] Siddiqui S.M. (2004). Role of the processing pore of the ClpX AAA+ ATPase in the recognition and engagement of specific protein substrates. Genes Dev..

[17] Martin A., Baker T.A., Sauer R.T. (2008). Pore loops of the AAA+ ClpX machine grip substrates to drive translocation and unfolding. Nat. Struct. Mol. Biol..

[18] Iosefson O., Nager A.R., Baker T.A., Sauer R.T. (2015). Coordinated gripping of substrate by subunits of a AAA+ proteolytic machine. Nat. Chem. Biol..

[19] Bell T.A., Baker T.A., Sauer R.T. (2019). Interactions between a subset of substrate side chains and AAA+ motor pore loops determine grip during protein unfolding. eLife.

[20] Tian L., Holmgren R.A., Matouschek A. (2005). A conserved processing mechanism regulates the activity of transcription factors Cubitus interruptus and NF-κB. Nat. Struct. Mol. Biol..

[21] Hoyt M.A., Zich J., Takeuchi J., Zhang M., Govaerts C., Coffino P. (2006). Glycine–alanine repeats impair proper substrate unfolding by the proteasome. EMBO J..

[22] Kraut D.A., Israeli E., Schrader E.K., Patil A., Nakai K., Nanavati D. (2012). Sequence- and species-dependence of proteasomal processivity. ACS Chem. Biol..

[23] Nassif N.D., Cambray S.E., Kraut D.A. (2014). Slipping up: partial substrate degradation by ATP-dependent proteases: partial substrate degradation by ATP-dependent proteases. IUBMB Life.

[24] Too P.H.-M., Erales J., Simen J.D., Marjanovic A., Coffino P. (2013). Slippery substrates impair function of a bacterial protease ATPase by unbalancing translocation *versus* exit. J. Biol. Chem..

[25] Kraut D.A. (2013). Slippery substrates impair ATP-dependent protease function by slowing unfolding. J. Biol. Chem..

[26] Levitskaya J., Sharipo A., Leonchiks A., Ciechanover A., Masucci M.G. (1997). Inhibition of ubiquitin/proteasome-dependent protein degradation by the Gly-Ala repeat domain of the Epstein–Barr virus nuclear antigen 1. Proc. Natl. Acad. Sci. U. S. A..

[27] Martin A., Baker T.A., Sauer R.T. (2005). Rebuilt AAA + motors reveal operating principles for ATP-fuelled machines. Nature.

[28] Hua B., Han K.Y., Zhou R., Kim H., Shi X., Abeysirigunawardena S.C. (2014). An improved surface passivation method for single-molecule studies. Nat. Met..

[29] Roche E.D. (1999). SsrA-mediated peptide tagging caused by rare codons and tRNA scarcity. EMBO J..

[30] Gottesman S., Roche E., Zhou Y., Sauer R.T. (1998). The ClpXP and ClpAP proteases degrade proteins with carboxy-terminal peptide tails added by the SsrA-tagging system. Genes Dev..

[31] Koodathingal P., Jaffe N.E., Kraut D.A., Prakash S., Fishbain S., Herman C. (2009). ATP-dependent proteases differ substantially in their ability to unfold globular proteins. J. Biol. Chem..

[32] Olivares A.O., Kotamarthi H.C., Stein B.J., Sauer R.T., Baker T.A. (2017). Effect of directional pulling on mechanical protein degradation by ATP-dependent proteolytic machines. Proc. Natl. Acad. Sci. U. S. A..

[33] Falk K., Gratama J.W., Rowe M., Zou J.Z., Khanim F., Young L.S. (1995). The role of repetitive DNA sequences in the size variation of Epstein--Barr virus (EBV) nuclear antigens, and the identification of different EBV isolates using RFLP and PCR analysis. J. Gen. Virol..

[34] Wootton J.C., Federhen S. (1996). Analysis of compositionally biased regions in sequence databases. Met. Enzymol..

[35] Wootton J.C., Federhen S. (1993). Statistics of local complexity in amino acid sequences and sequence databases. Comput. Chem..

[36] Li H., Carrion-Vazquez M., Oberhauser A.F., Marszalek P.E., Fernandez J.M. (2000). Point mutations alter the mechanical stability of immunoglobulin modules. Nat. Struct. Biol..

[37] Henderson A., Erales J., Hoyt M.A., Coffino P. (2011). Dependence of proteasome processing rate on substrate unfolding. J. Biol. Chem..

[38] Joshi S.A., Hersch G.L., Baker T.A., Sauer R.T. (2004). Communication between ClpX and ClpP during substrate processing and degradation. Nat. Struct. Mol. Biol..

[39] Jonsson E., Htet Z.M., Bard J.A.M., Dong K.C., Martin A. (2021). Ubiquitin modulates 26S proteasome conformational dynamics and promotes substrate degradation. bioRxiv.

[40] Saunders R.A., Stinson B.M., Baker T.A., Sauer R.T. (2020). Multistep substrate binding and engagement by the AAA+ ClpXP protease. Proc. Natl. Acad. Sci. U. S. A..

[41] Kenniston J.A., Baker T.A., Sauer R.T. (2005). Partitioning between unfolding and release of native domains during ClpXP degradation determines substrate selectivity and partial processing. Proc. Natl. Acad. Sci. U. S. A..

[42] Zhang M., Coffino P. (2004). Repeat sequence of epstein-barr virus-encoded nuclear antigen 1 protein interrupts proteasome substrate processing. J. Biol. Chem..

[43] Freibaum B.D., Taylor J.P. (2017). The role of dipeptide repeats in C9ORF72-related ALS-FTD. Front. Mol. Neurosci..

[44] Rodriguez-Aliaga P., Ramirez L., Kim F., Bustamante C., Martin A. (2016). Substrate-translocating loops regulate mechanochemical coupling and power production in AAA+ protease ClpXP. Nat. Struct. Mol. Biol..

[45] Bard J.A.M., Bashore C., Dong K.C., Martin A. (2019). The 26S proteasome utilizes a kinetic gateway to prioritize substrate degradation. Cell.

[46] Chang Y.-J., Jeng U.-S., Chiang Y.-L., Hwang I.-S., Chen Y.-R. (2016). The glycine-alanine dipeptide repeat from C9orf72 hexanucleotide expansions forms toxic amyloids possessing cell-to-cell transmission properties. J. Biol. Chem..

[47] Guo Q., Lehmer C., Martínez-Sánchez A., Rudack T., Beck F., Hartmann H. (2018). *In situ* structure of neuronal C9orf72 poly-GA aggregates reveals proteasome recruitment. Cell.

[48] Yu H., Singh Gautam A.K., Wilmington S.R., Wylie D., Martinez-Fonts K., Kago G. (2016). Conserved sequence preferences contribute to substrate recognition by the proteasome. J. Biol. Chem..

[49] Lin L., Ghosh S. (1996). A glycine-rich region in NF-κB p105 functions as a processing signal for the generation of the p50 subunit. Mol. Cell Biol..

[50] Pan Y., Wang B. (2007). A novel protein-processing domain in Gli2 and Gli3 differentially blocks complete protein degradation by the proteasome. J. Biol. Chem..

[51] Schrader E.K., Harstad K.G., Holmgren R.A., Matouschek A. (2011). A three-part signal governs differential processing of Gli1 and Gli3 proteins by the proteasome. J. Biol. Chem..

[52] Lane C.A., Hardy J., Schott J.M. (2018). Alzheimer’s disease. Eur. J. Neurol..

[53] Saudou F., Humbert S. (2016). The biology of huntingtin. Neuron.

[54] Antony P.M.A., Diederich N.J., Krüger R., Balling R. (2013). The hallmarks of Parkinson’s disease. FEBS J..

[55] Roy R., Hohng S., Ha T. (2008). A practical guide to single-molecule FRET. Nat. Met..

[56] Johnson D.S., Jaiswal J.K., Simon S. (2012). Total internal reflection fluorescence (TIRF) microscopy illuminator for improved imaging of cell surface events. Curr. Protoc. Cytometry.

[57] van der Walt S., Schönberger J.L., Nunez-Iglesias J., Boulogne F., Warner J.D., Yager N. (2014). scikit-image: image processing in Python. PeerJ.

