# Supplementary material for: Single molecule microscopy reveals diverse actions of substrate sequences that impair ClpX AAA+ ATPase function
Source: J Biol Chem. 2022 Sep 5;298(10):102457. doi: 10.1016/j.jbc.2022.102457 (PMC9531181; doi:10.1016/j.jbc.2022.102457)
Supplement: Supporting Information [file mmc1.docx]

**Single-molecule microscopy reveals diverse actions of substrate sequences that impair ClpX AAA+ ATPase function**

Xiao Wang^1^, Sanford M. Simon^1^, Philip Coffino^1^*

# Supporting Information

**Figure S1:** Assessment of coverslip passivation using Alexa488-streptavidin.

**Figure S2:** Testing ClpX-dependent immobilization of ssrA-tagged substrate in TIRF microscopy.

**Figure S3:** Testing the specific capture of ssrA-tagged substrates by immobilized ClpX using an ssrADD control substrate in TIRF microscopy.

**Figure S4:** TIRF data processing flow chart for constructing a single trace from puncta collected in time-lapse movies.

**Figure S5:** Representative trajectories of substrate binding events, recorded by TIRF microscopy

**Figure S6:** Diagram showing possible engagement of the N terminus of an ssrA-tagged substrate by ClpX.

**Figure S7:** Full length cpGFP-ecDHFR is resistant to ClpXP degradation when DHFR stabilized by MTX.

**Figure S8:** Photobleaching is not a limiting factor for the measurement of the dwell time distributions under the conditions used for the experiments.

**Figure S9:** Average dwell time (τ) plotted against sequence complexity for all test sequences.

**Table S1:** calculation of sequence complexity using two methods


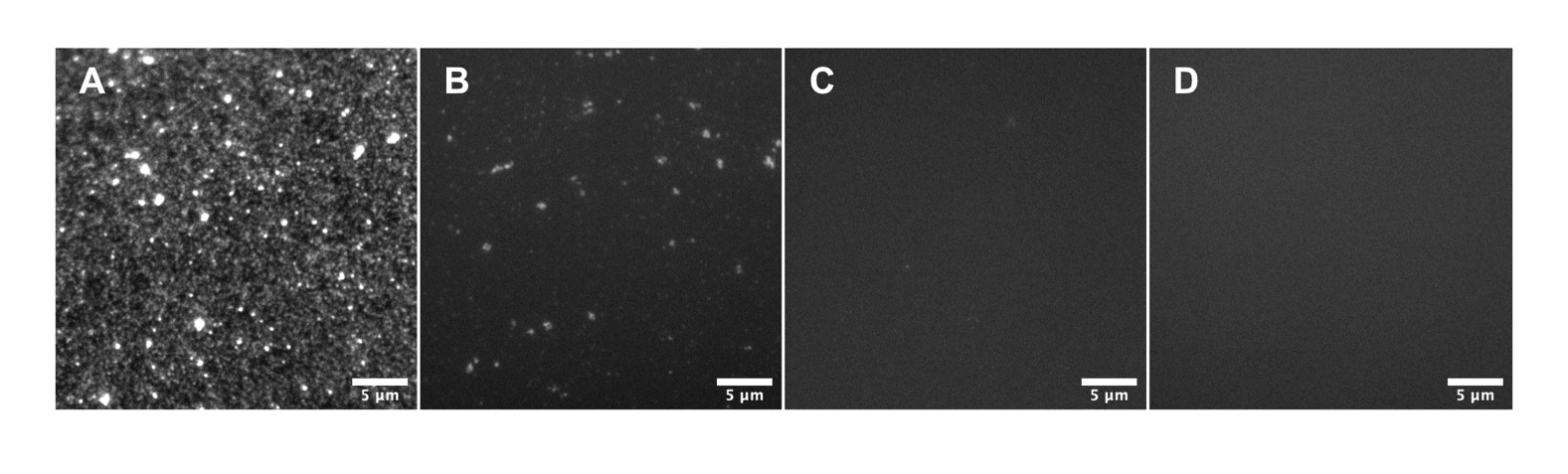


**Figure S1: Assessment of coverslip passivation using Alexa488-streptavidin.** *A*, the DDS-Tween-20 passivated coverslip was treated with 0.2 mg/mL biotinylated BSA, then incubated with 0.01 mg/mL Alexa-488 labeled streptavidin (~170 nM) and examined for immobilized streptavidin under TIRF illumination with 488 nm laser for excitation. The coverslip was densely populated with Alexa488-streptavdin puncta. *B*, same condition as in A, except that 2.0 µg/mL biotinylated BSA was used to treat the coverslip. Puncta density is sharply reduced as a result. *C*, same condition as in A, except that 0.2 µg/mL of biotinylated BSA was used to treat the coverslip. Puncta density was further reduced compared to B. *D*, same condition as in A, except that 0.2 mg/mL unmodified BSA was used to treat the coverslip. Nonspecific binding of Alexa488-streptavidin to the coverslip was not evident.


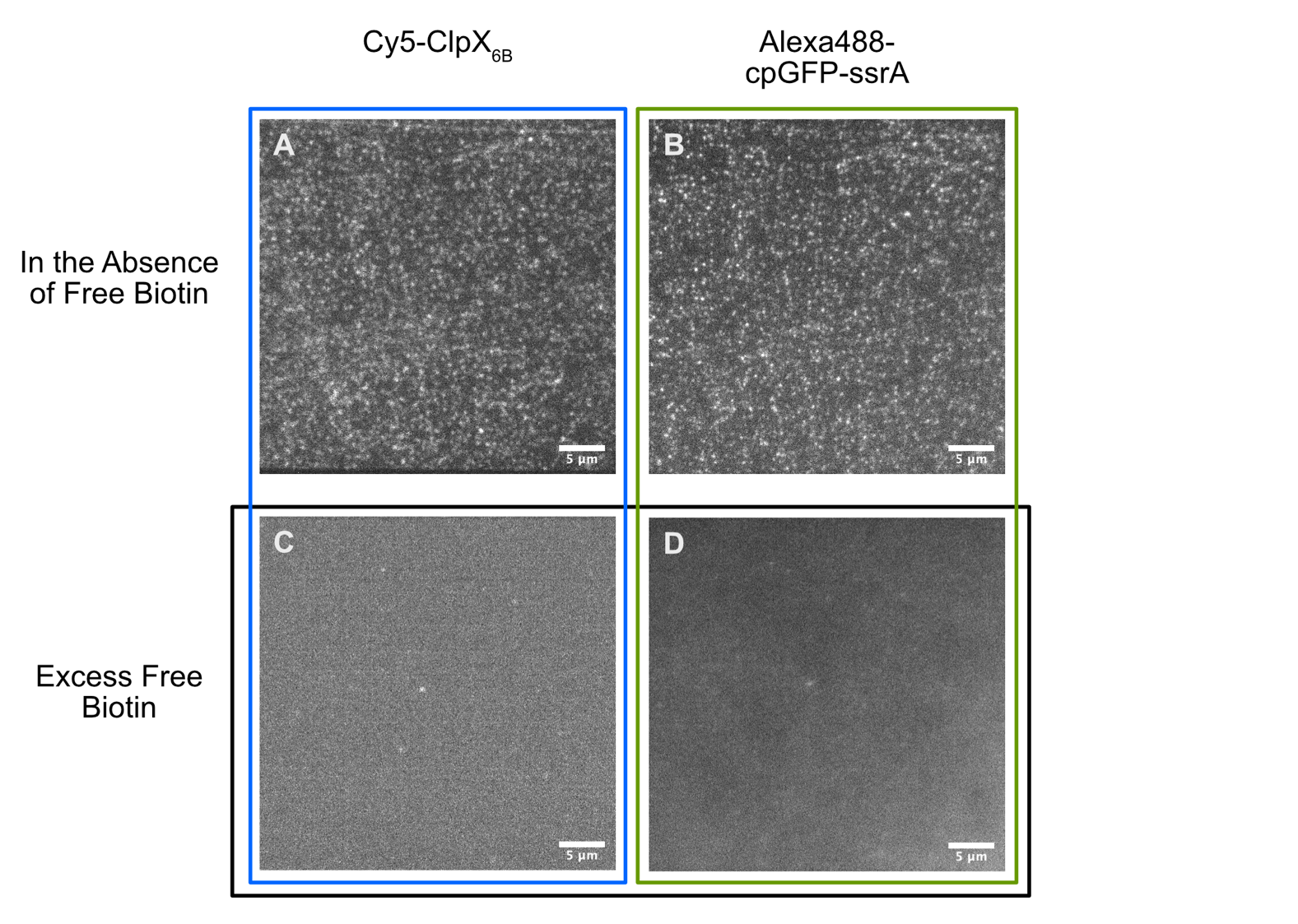


**Figure S2: Testing ClpX-dependent immobilization of ssrA-tagged substrate in TIRF microscopy**. *A-B*, ClpX (50 nM) was immobilized via biotinylated-BSA-streptavidin anchors on the coverslip, as shown in **Figure 1B** and demonstrated in **Figure S1**. *A*, puncta of immobilized Cy5-ClpX6B under TIRF illumination. ClpX6B were labeled at the native cysteine residue with thiol-reactive Cy5 maleimide dye, and were illuminated by 647 nm laser. *B*, puncta of Alexa-488 labeled cpGFP-ssrA (50 nM) under TIRF illumination at the same field-of-view as in A; cpGFP-ssrA were labeled using thiol-reactive Alexa488 maleimide. Labeling reactions were carried out under the same condition as labeling of cpGFP-DHFR substrates using cy3 or cy5, as described in Methods. Fluorophore was illuminated by 488 nm laser. *C-D*, As in A and B, but 1 mM free biotin were used to occupy biotin sites on immobilized streptavidin before the introduction of ClpX and substrates. *C*, imaging of Cy5-ClpX6B. Puncta density was highly reduced by biotin competition. *D*, Alexa488-cpGFP-ssrA could not be immobilized without immobilization of ClpX6B.


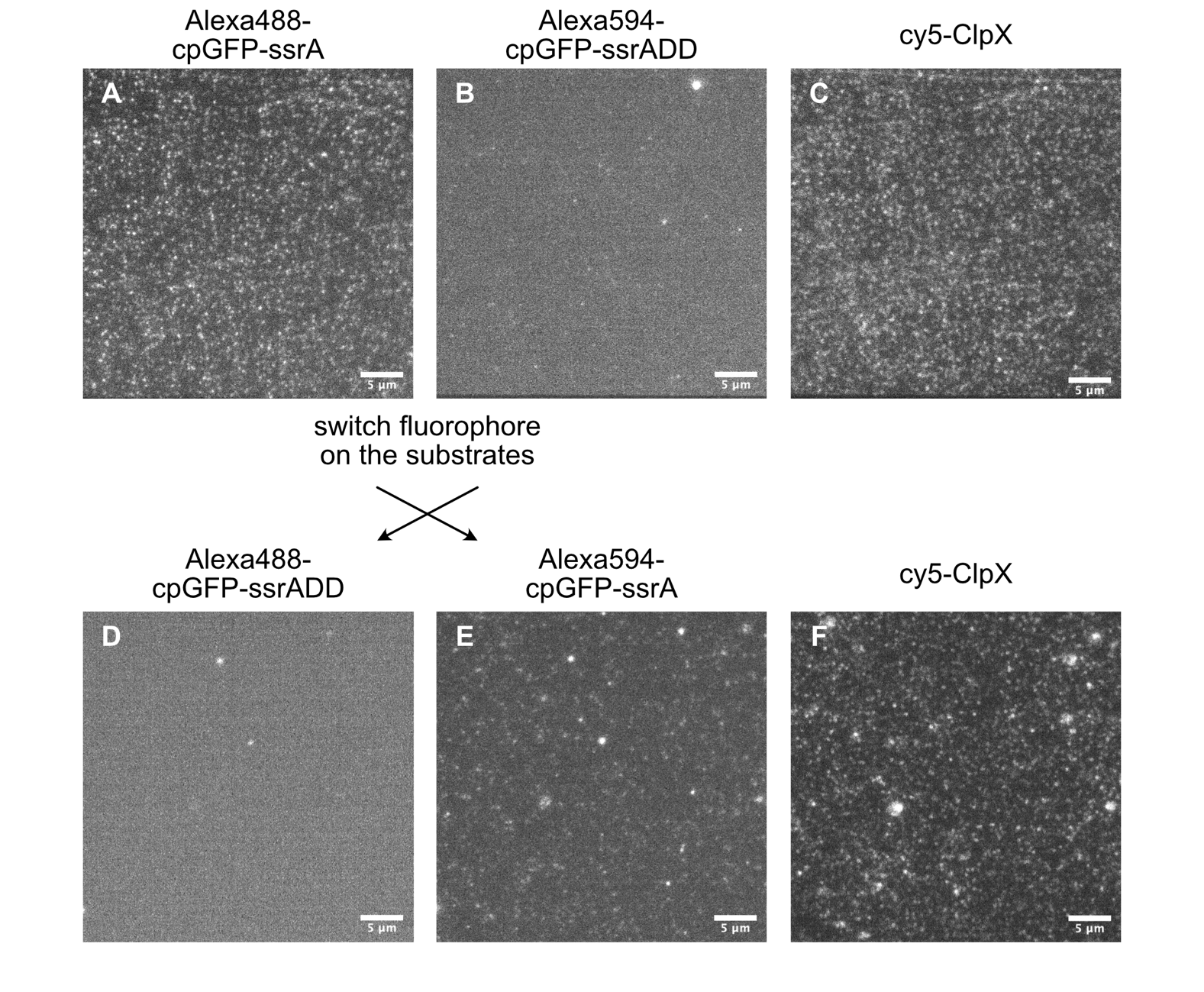


**Figure S3: Testing the specific capture of ssrA-tagged substrates by immobilized ClpX using an ssrADD control substrate in TIRF microscopy.** *A-C*, same condition as in SI Fig 2A-2B, but with the addition of 50 nM of Alexa594-cpGFP-ssrADD. *A,* puncta of Alexa488 labeled cpGFP-ssrA (50 nM) under TIRF illumination with 488 nm laser. *B,* puncta of Alexa594 labeled cpGFP-ssrADD (50 nM) in the same field-of-view as A under TIRF illumination; cpGFP-ssrADD were labeled using thiol-reactive Alexa594, with 543 nm laser. *C,* puncta of immobilized Cy5-ClpX6B under TIRF illumination in the same field-of-view as A. *D-F,* same condition as in A-C, except that cpGFP-ssrA was labeled by Alexa594 and cpGFP-ssrADD was labeled by Alexa488. *D,* puncta of Alexa488 labeled cpGFP-ssrADD (50 nM) under TIRF illumination with 488 nm laser. *E,* puncta of Alexa594 labeled cpGFP-ssrA (50nM) under TIRF illumination in the same field-of-view as D, excited by 543 nm laser. *F,* puncta of cy5-ClpX6B under TIRF illumination in the same field-of-view as D, excluded by 647 nm laser.


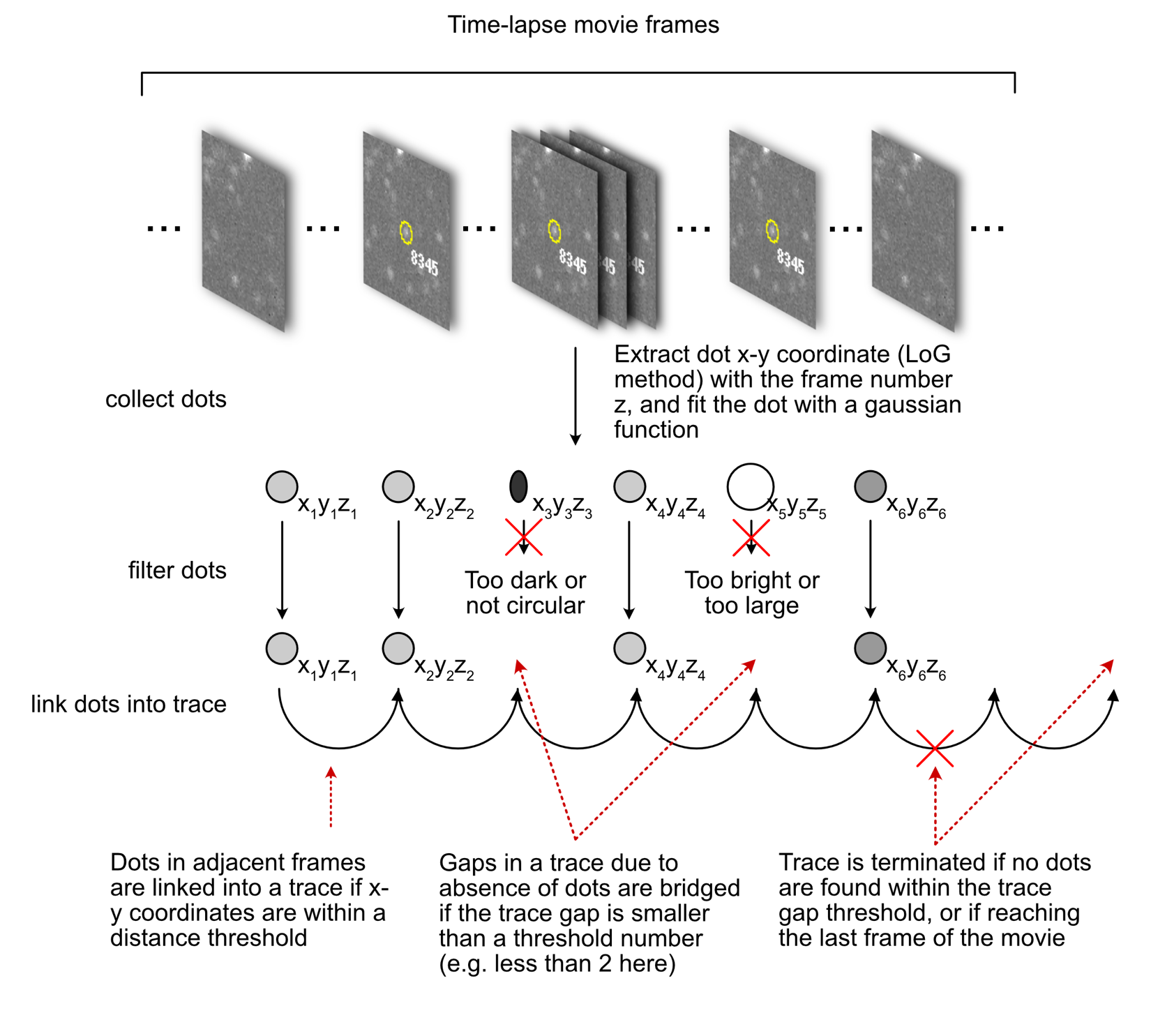


**Figure S4: TIRF data processing flow chart for constructing a single trace from dots collected in time-lapse movies.** The data processing is done in three steps. First, dots on each frame of the movie are identified using a published Laplacian of Gaussian method, which returns the x-y coordinates of the dot. Next, all dots collected are first fitted with a 2D gaussian function, the parameters of which are used to filter out dots with irregular shapes and brightness. Finally, dots are linked as traces and serve as nodes in the path, according to the proximity of their x-y coordinates. The length of the trace, measured in the number of frames it spans, thus reflects the dwell time of the punctum.


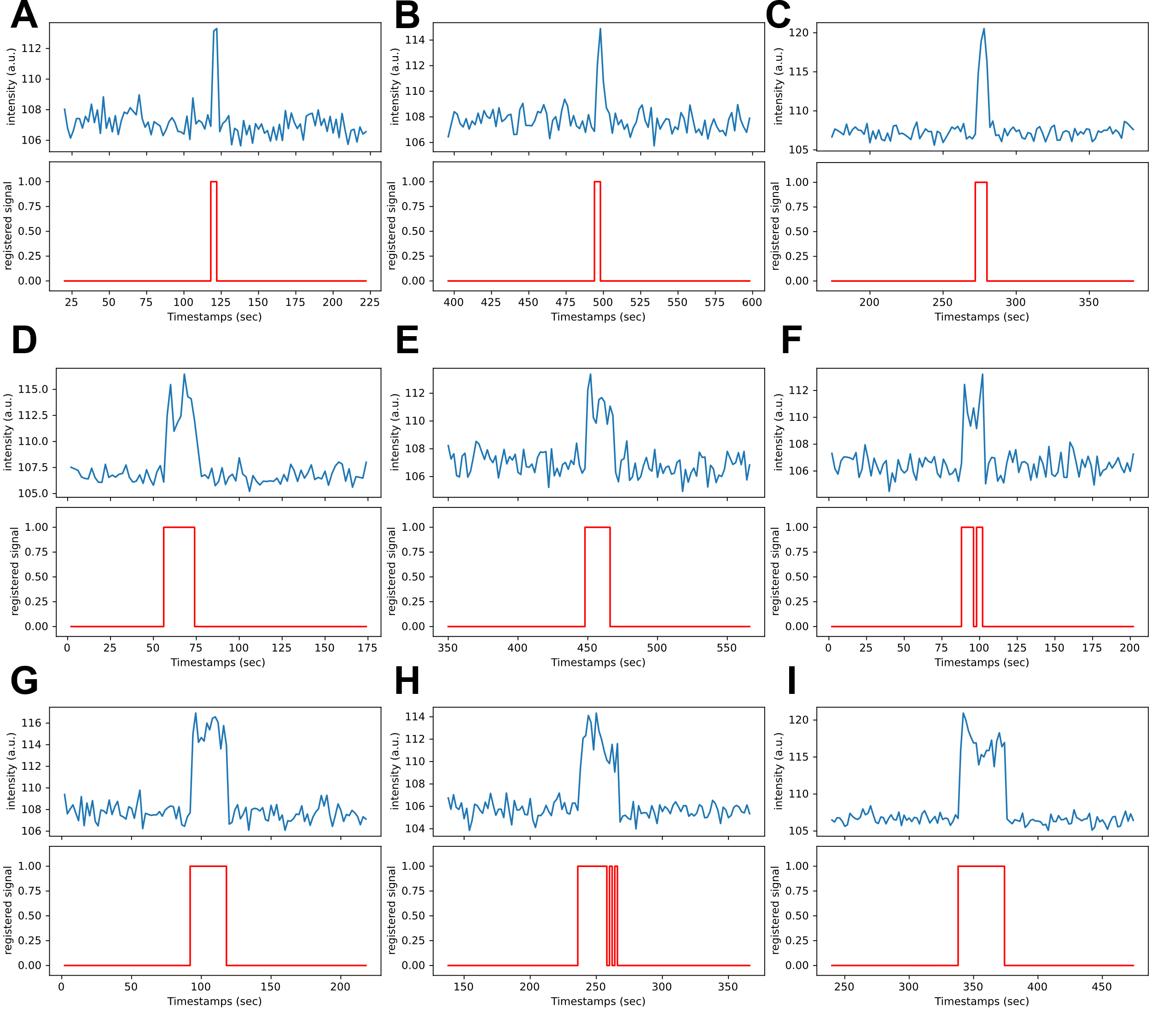


**Figure S5: Representative trajectories of substrate binding events, recorded by TIRF microscopy.** For each trajectory, the top panel shows the mean intensity over time, while the bottom panel shows whether the data processing script registered a punctum at the corresponding frame. *A-C*, representative traces for events under 10 seconds. *D-F*, representative traces for events between 10-20 seconds. *G-I*, representative traces for events greater than 20 seconds. The gaps in the processed trajectories (*F, H)* were bridged using the process described in Figure S4.


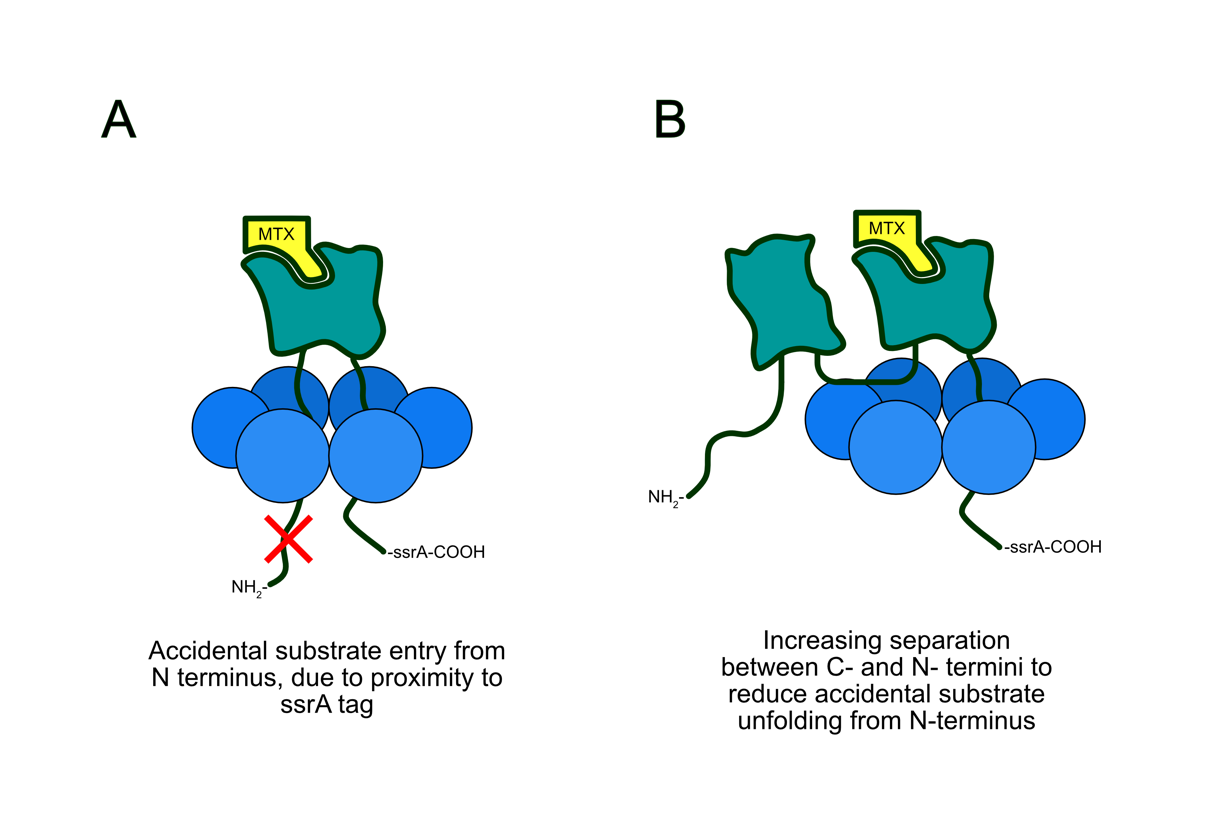


**Figure S6: Possible engagement of the N terminus of an ssrA-tagged substrate by ClpX.** *A*, the proximity of the N and C-terminus on a small substrate might allow simultaneous engagement of both termini by the ClpX central pore. *B*, adding another domain at the N-terminal side of the DHFR domain may impair N-terminal entry, and allow detection of unfolding from the N-terminus by selective degradation of the N-terminal cpGFP domain.


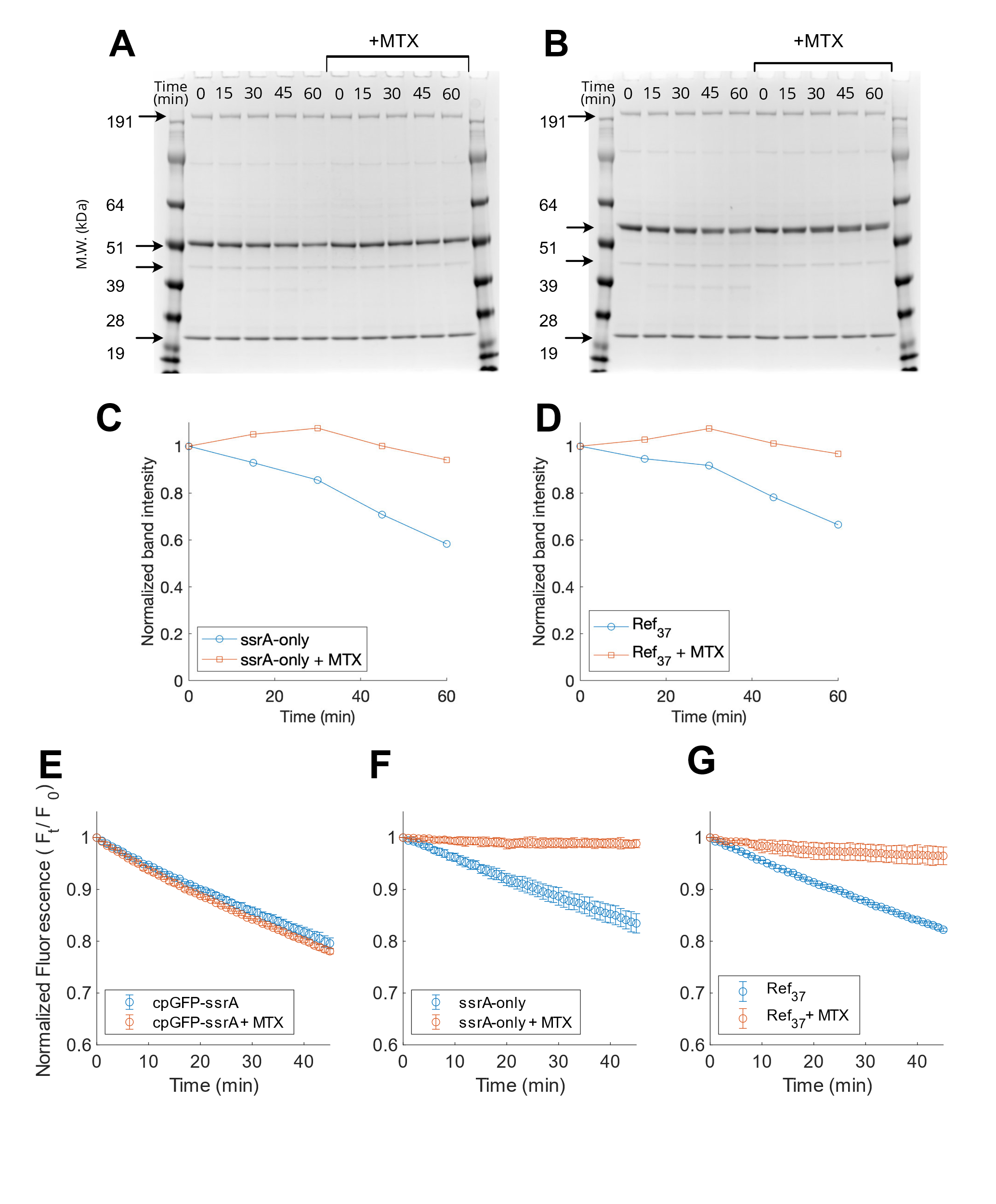


**Figure S7: Full length cpGFP-ecDHFR is resistant to ClpXP degradation when DHFR stabilized by MTX.** *A*, Coomassie staining of an SDS-PAGE gel monitoring the time-dependent degradation of 2 µM cpGFP-DHFR-ssrA substrate by 100 nM ClpXP at 2 mM ATP. The substrate has only the ssrA tag as its C-terminal tail. Incubation was for 1 hour at 30 °C, in the absence or presence of 1 mM MTX. The arrows indicate four major bands, which corresponds to the four proteins present in the reaction—ClpX6B (248 kDa) , cpGFP-DHFR-ssrA substrate (51.2 kDa), Creatine phosphokinase monomer (40 kDa), ClpP monomer (24.3 kDa). *B*, degradation of cpGFP-DHFR-Ref_37_-ssrA (54.1 kDa) by ClpXP under the same condition as A. The tail extension sequence (Ref_37_) is described in Figure 2A. *C*, quantification of the bands corresponding to the substrates in A. *D*, quantification of the bands corresponding to the substrates in B. *E-G*, degradation of 2.0 µM ssrA-tagged substrates by 100 nM ClpXP at 30 °C with 2 mM ATP, monitored by cpGFP fluorescence intensity over time. Data are shown as mean ± S.D. for three biological replicates. *E*, degradation of cpGFP-ssrA by ClpXP. The presence of MTX does not inhibit ClpXP activity. *F*, degradation of cpGFP-DHFR-ssrA by ClpXP. *G*, degradation of cpGFP-DHFR-Ref_37_-ssrA by ClpXP.


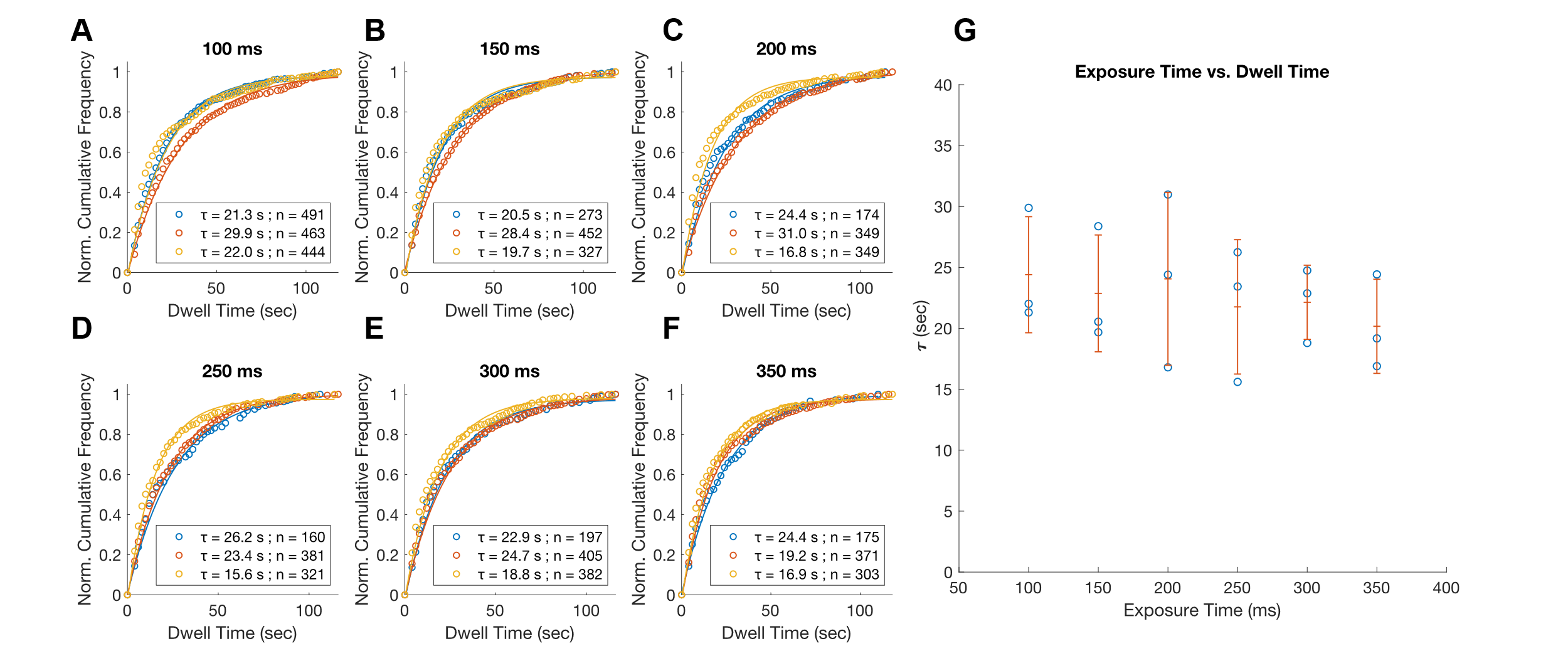


**Figure S8, photobleaching is not a limiting factor for the measurement of the dwell time distributions under the conditions used for the experiments.** The dwell time of a substrate (DHFR with Ref_37_ tail, which has long dwell time with ClpX, as shown in **Figure 2**) was measured using different exposure times under the same laser output and time interval. *A-F*: dwell time distributions of the three biological replicates, measured under 5 mW 561nm laser output with exposure time per frame ranging from 100 ms to 350 ms, and fitted with r^2^ > 0.95. *G*, average dwell time (derived from A-F) plotted against the corresponding exposure time, with 50 ms increment; bars represent mean ± S.D. A, 100 ms exposure. B, 150 ms exposure. C, 200 ms exposure. D, 250 ms exposure. E, 300 ms exposure. F, 350 ms exposure.


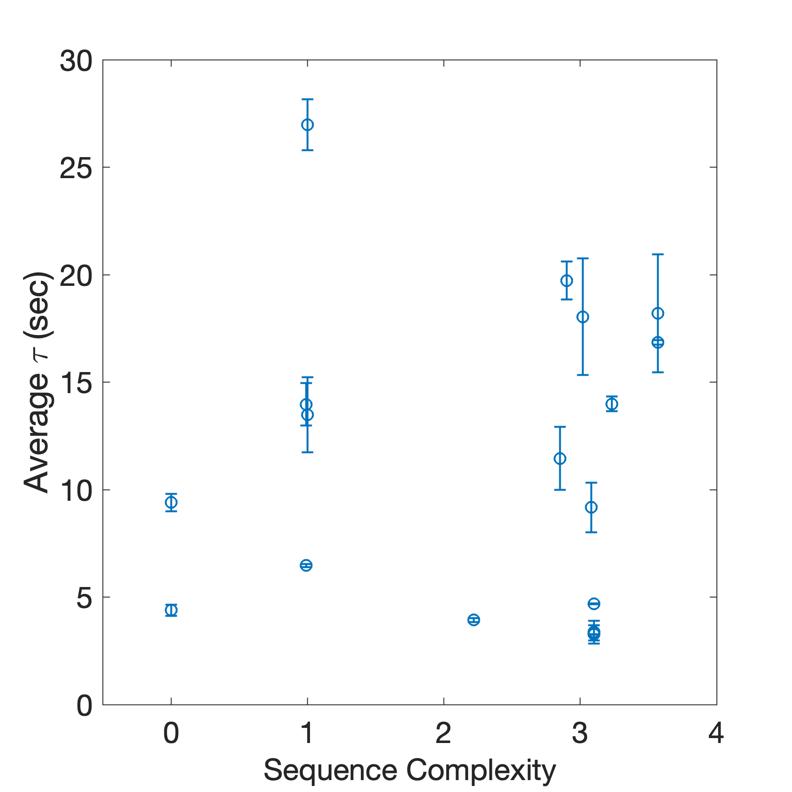


Figure S9, average dwell time (τ) plotted against sequence complexity for all test sequences. For all test sequences, the corresponding dwell times from TIRF measurements in DHFR substrates were plotted against their sequence complexity scores. Error bar represents the standard deviation of dwell time measurements between replicates. The complexity scores calculated here are based on the Shannon’s definition for information entropy.

| Motif Name | Sequence | Complexity K1 (Boltzmann Entropy) | Complexity K2 (Shannon's Informational Entropy) |
| --- | --- | --- | --- |
| Ref_11_ | GLGARSAGITH | 1.97 | 2.85 |
| Ref_22_ | GLGARSAGITHLERPHRGLGDI | 2.49 | 3.23 |
| Ref_37_ | GLGARSAGITHLERPHRGLGDISDQEAKPSTEDLGDK | 2.95 | 3.57 |
| Ref_74_ | GLGARSAGITHLERPHRGLGDISDQEAKPSTEDLGDKGLGARSAGITHLERPHRGLGDISDQEAKPSTEDLGDK | 3.19 | 3.57 |
| ssrA | AANDENYALAA | 1.58 | 2.22 |
| polyG_11_ | GGGGGGGGGGG | 0 | 0 |
| polyG_11_^var1^ | GGGGGGGGGGGLERPHRGLGDISDQEAKPSTEDLGDK | 2.52 | 3.08 |
| polyG_11_^var12^ | GLGARSAGITHGGGGGGGGGGGSDQEAKPSTEDLGDK | 2.47 | 3.02 |
| polyG_11_^var27^ | GLGARSAGITHLERPHRGLGDISDQEGGGGGGGGGGG | 2.38 | 2.90 |
| GA_11_ | AGAGGAAGAGG | 0.80 | 0.99 |
| GS_11_ | SGSGGSSGSGG | 0.80 | 0.99 |
| GA_37_ | AGAGGAAGAGGGGAAGAAAAAGAGAGAGGAGAAAGGG | 0.92 | 1.00 |
| GS_37_ | SGSGGSSGSGGGGSSGSSSSSGSGSGSGGSGSSSGGG | 0.92 | 1.00 |
| SUMO_11_ | AKPSTEDLGDK | 2.11 | 3.10 |
| SUMO_11_^PY^ | AKYSTEDLGDK | 2.11 | 3.10 |
| SUMO_11_^SCR^ | KAGLSTPEDKD | 2.11 | 3.10 |
| SUMO_11_^REV^ | KDGLDETSPKA | 2.11 | 3.10 |
| polyS_11_ | SSSSSSSSSSS | 0 | 0 |

**Table S1: calculation of sequence complexity using two methods**
